# Assessment of the Validity and Quality of Polycystic Ovarian Syndrome (PCOS) Screening Tools Available for Women Globally: A Systematic Review

**DOI:** 10.3390/clinpract14050131

**Published:** 2024-08-23

**Authors:** Lea Sacca, Diana Lobaina, Elisheva Knopf, Sara Burgoa, Samantha Jimenez, Goodness Okwaraji, Madison Etzel, Vartiter Vardanyan, Madison Tharp, Meera Rao, Vama Jhumkhawala, Joshua Sohmer, Sebastian Densley, Niko Linzer, Pranav Meka, Daniella Diaz, Michelle Knecht, Dawn Kimberly Hopkins, Panagiota Kitsantas, Maria Mejia, Candy Wilson

**Affiliations:** 1 Department of Population Health and Social Medicine, Charles E. Schmidt College of Medicine, Florida Atlantic University, Boca Raton, FL 33431, USA; dlobaina2021@health.fau.edu (D.L.); eknopf2023@health.fau.edu (E.K.); sburgoa2022@health.fau.edu (S.B.); jimenezs2023@health.fau.edu (S.J.); gokwaraji2018@health.fau.edu (G.O.); metzel2023@health.fau.edu (M.E.); vvardanyan2012@health.fau.edu (V.V.); mtharp2022@health.fau.edu (M.T.); mrao2022@health.fau.edu (M.R.); vjhumkhawala2022@health.fau.edu (V.J.); jsohmer2022@health.fau.edu (J.S.); sdensley2022@health.fau.edu (S.D.); nlinzer2022@health.fau.edu (N.L.); pmeka2019@health.fau.edu (P.M.); kebam@health.fau.edu (M.K.); pkitsanta@health.fau.edu (P.K.); mejiam@health.fau.edu (M.M.); 2 Charles E. Schmidt College of Science, Boca Raton, FL 33431, USA; diazd2019@fau.edu; 3 Henry M. Jackson Foundation for the Advancement of Military Medicine, Bethesda, MD 20817, USA; dawn-kimberly.hopkins.ctr@usuhs.edu; 4 Christine E. Lynn College of Nursing, Florida Atlantic University, Boca Raton, FL 33431, USA; candywilson@health.fau.edu

**Keywords:** polycystic ovary syndrome, PCOS, diagnosis, mental health, chronic pain, screening tools, women’s health, systematic review

## Abstract

**Background:** This systematic review has the following aims: (1) to identify measurement tools used globally by healthcare providers to diagnose PCOS in women at elevated risk; (2) to assess the comprehensiveness of these tools regarding mental health and chronic pain; (3) to list strategies for validating, disseminating, and implementing these tools; and (4) to provide future recommendations for experts in healthcare settings. **Methods:** This review utilized the Preferred Reporting Items for Systematic reviews and Meta-Analyses (PRISMA) and the Arksey and O’Malley York methodology. Studies were sourced from the PubMed, Embase, and Cochrane Library databases, with inclusion criteria focusing on peer-reviewed articles addressing PCOS diagnosis and associated comorbidities. Data extraction and analysis followed the Joanna Briggs Institute (JBI) recommendations. **Results:** A total of 63 studies met the inclusion criteria. Findings indicate that current screening tools for PCOS often lack comprehensive integration of mental health and chronic pain assessments. Tools like the PCOSQ and its updated version, PCOSQ-50, inadequately address pain-related symptoms, highlighting a gap in holistic patient evaluation. This review identified significant associations between PCOS and mental health disorders, including anxiety and depression, emphasizing the need for mental health screenings as part of PCOS management. **Conclusions:** There is a critical need for validated PCOS screening tools that encompass both physical and psychological aspects of the condition. Educating healthcare providers on the cultural and social determinants influencing PCOS can improve diagnosis and patient outcomes. Future research should focus on developing holistic screening tools and culturally relevant educational resources, aiming to enhance the overall quality of life for women with PCOS.

## 1. Background

Polycystic ovary syndrome (PCOS) is a complex disease affecting primarily women of reproductive age [1]. An estimated 8 to 13 percent of women of reproductive age are affected by PCOS worldwide (Refs. [2,3]), yet up to 70% of these women remain undiagnosed [2]. Changes associated with PCOS, including obesity, hirsutism, and menstrual irregularities, contribute to significant psychological challenges, increasing the risk for anxiety, depression, and suicide [4,5]. Anxiety and depression are particularly prevalent (Refs. [6,7]), with studies showing significantly higher rates in women with PCOS compared with those without the condition [7,8]. Early diagnosis and treatment of PCOS can mitigate not only the physical manifestations of PCOS but also help in managing the psychological burden of the disease [5,9]. It also allows for a more comprehensive treatment approach that includes psychological support, lifestyle modifications, and medical management [5,9,10]. Early treatment can also prevent long-term complications such as type 2 diabetes, cardiovascular diseases, and endometrial cancer [5,9,10]. However, the impact on mental health often remains a neglected aspect of these complications, despite its significant influence on a woman’s quality of life and overall wellbeing [5,8].

Despite the large prevalence of PCOS globally, diagnosis is a continuaous challenge for providers. PCOS is difficult to attribute to a single diagnostic test due to the complex and variable nature of its pathogenesis [11,12]. The Rotterdam diagnostic criteria are a globally utilized set of criteria that encompasses three features, oligovulation or anovulation; clinical or biochemical hyperandrogenism; and polycystic ovaries [13]. Two of three of these criteria must be met to be diagnosed with PCOS based on the current recommended guideline for diagnosis of PCOS [11,13]. However, it is important to note that PCOS is a condition that typically changes over time, especially during menarche, and thus these criteria may not always be met [12,13]. Additionally, there are various metabolic characteristics of PCOS that are not encompassed by these criteria but are still recognized as a significant part of this condition [14]. Overall, the fluctuating nature of PCOS and the incomplete understanding of its etiology makes it difficult to diagnose.

Given the variance in PCOS diagnostics and impact, it is evident that there remains a need to examine PCOS more closely. Particularly, there is a need to understand how healthcare providers identify it, whether existing diagnostic tools are feasible for use, and whether they include the various effects and challenges that are faced by women with PCOS. The purpose of this study is four-fold. First, we seek to determine which measurement tools have been utilized by healthcare providers globally to diagnose PCOS in at-risk women. Second, we aim to determine the extent to which mental health issues and chronic pain symptoms are included in these tools. Third, we seek to assess which strategies have been adopted to validate, disseminate, and implement such tools. Finally, this paper will provide future recommendations for experts adopting and adapting such tools in healthcare settings.

## 2. Methods

The review team consisted of a team of medical students, public health experts, and nursing professionals with extensive knowledge regarding the impact of PCOS on adolescent and adult women globally. The Preferred Reporting Items for Systematic reviews and Meta-Analyses (PRISMA) was utilized as a reference checklist in writing the study sections. The Arksey and O’Malley York methodology was used as guidance for this review and consists of five steps: (1) identifying research questions; (2) searching for relevant studies; (3) selecting studies relevant to the research questions; (4) charting the data; and (5) collating, summarizing, and reporting results. The Joanna Briggs Institute (JBI) recommendations were also used for the extraction, analysis, and presentation of results in this scoping review. These methods ensure transparency, permit replicability of the search strategy, and increase the reliability of this study’s findings [15].

**Step 1: identify research questions.**

Four research questions were used for this scoping review: (1) Which measurement tools have been utilized by healthcare providers globally to diagnose PCOS in at-risk women? (2) To what extent are mental health issues and chronic pain symptoms included in these tools? (3) Which strategies have been adopted to validate, disseminate, and implement such tools? (4) What are future recommendations for experts adopting and adapting such tools in healthcare settings?

**Step 2: search for relevant studies.**

Keywords and Medical Subject Headings (MeSH) terms were created by the senior research librarian (MK), who is an expert in systematic review protocols. Search terms included the following: *PCOS*, *surveys*, *questionnaires*, *screening tools*, *diagnosis*, *quality*, *women*, *health providers*, *physicians*, *primary care*, *obstetrics and gynecology*, *endocrinology*, *female adolescents*, and *female adults*. The Rayyan platform, an online interactive platform for researchers to develop systematic and scoping reviews, was used to condense all articles generated from our search. Three electronic databases (PubMed, Embase, Cochrane Library) were searched to identify the peer-reviewed literature from primary data sources, secondary data sources, and case reports. The review of the literature was completed over a period of 4 months, from November 2023 to February 2024. The screening of these articles was carried out by the senior author and coauthors (DL, GO, JS, SD, MT, VV, EK, SJ, ME, NL, PM, DD).

### 2.1. Inclusion

The articles that were included were peer-reviewed studies published in English between 2000 and 2023 that addressed PCOS diagnosis in adolescent and adult women globally. These studies included measurement and screening tools for PCOS diagnosis. Studies including screening tools for PCOS-associated comorbidities such as mental health and chronic pain were also included.

### 2.2. Exclusion

Excluded studies encompassed systematic, scoping, narrative, and literature reviews as well as published abstracts. Additionally, articles were excluded if they did not include screening tools for PCOS, focused on general screening tools for different reproductive health issues rather than focusing on only PCOS, or were surveys measuring knowledge of and perceptions toward PCOS. An initial screening of the articles after extraction from relevant databases and the construction of the Rayyan page specific to this study was conducted by lead author. Reviewers (DL, GO, JS, SD, MT, VV, EK, SJ, ME, NL, PM, DD) conducted secondary screening of the titles and abstracts. Disagreements were resolved by reaching consensus through discussions that involved the lead author.

**Step 3: selection of studies relevant to the research questions.**

All coauthors extracted, summarized, and tabulated data. The senior author reviewed all tabulated data to resolve any discrepancies for reliability and validity purposes. Summary tables included one evidence table describing study characteristics (Table S1) and one including type of methodology used to screen for PCOS in female adolescents and adults globally, whether the tool used was validated or not, the limitations of the measurement tool used, mode of tool administration, analysis used to measure associations between PCOS diagnosis and constructs of interest, and, when relevant, theoretical framework used to guide survey development (Table S2). Table 1 assesses the role of chronic pain and mental health disorders in PCOS diagnosis. Table 2 displays dissemination and implementation strategies for the widespread utility and application of PCOS screening tools. Table 3 is a lessons-learned table, where a basic qualitative content analysis was carried out to identify similar themes mentioned throughout the studies to guide future research directions. The final table consists of the application of the Critical Appraisal Skills Programme (CASP) checklist to assess study rigor and quality (Table 4).

**Steps 4 and 5: data charting and collation, summarization, and reporting of results.**

Study characteristics were tabulated for article number, primary author/year, country, study design, sample size, study population, age range, study purpose, constructs measured, type of social determinants of health (SDoH), and status of PCOS (Table S1). Table S2 is organized by primary author/year, type of methodology used in the study, whether it was validated, if there were any limitations for the measurement tool used, the mode of administration of tool, if there were any theoretical frameworks used, the analysis performed to look for associations between PCOS diagnosis and the constructs, and the variables analyzed to measure associations and whether those associations were significant. Table 1 includes information on primary author/year, measurement of mental health disorder, type of mental health disorders, number of mental health survey items, measurement of chronic pain, type of chronic pain, number of chronic pain survey items, and whether there was a significant association in any of the constructs. Table 2 is tabulated by article number, primary author/year, and strategies used for validation, dissemination, and implementation of PCOS screening tools. For Table 3, the three phases of qualitative content analysis for the results of primary qualitative research described by Elo and Kyngas (2008) are applied: (i) preparation, (ii) organizing, and (iii) reporting [16].

Lastly, the CASP checklist was applied to transparently appraise original research studies by providing a framework to access the credibility, relevance, strengths, and limitations of the results (Table 4) [17]. Two of the coauthors, VJ and MR, evaluated the rigor and quality of the studies using the Critical Appraisal Skills Programme (CASP) checklist [17]. This checklist has been utilized by previous scoping reviews [18]. The CASP checklist implemented in this scoping review encompasses the following criteria: (1) clarity of stated study aims and objectives, (2) appropriateness of study design, (3) adequate description of the methodology and subject selection, (4) potential bias in sample selection, (5) representativeness of the sample for generalizability of study results, (6) use of statistical power analysis for sample size calculation, (7) response rate, (8) use of reliable and valid measures, (9) examination for statistical significance, and (10) inclusion of confidence intervals (CI) in study findings [17,18]. The response to each criterion was classified as “Yes”, “No”, or “Unsure”, and a quality score for each study was derived by summing the number of “Yes” responses. However, for item 4, the reverse score “No” was counted within the rigor indices that contributed to the overall quality score of each included study [17,18]. The CASP checklist findings are presented in Table 4.

## 3. Registration

The study protocol for this systematic review was registered in PROSPERO (ID: CRD42024513854).

## 4. Results

**Figure 1:**
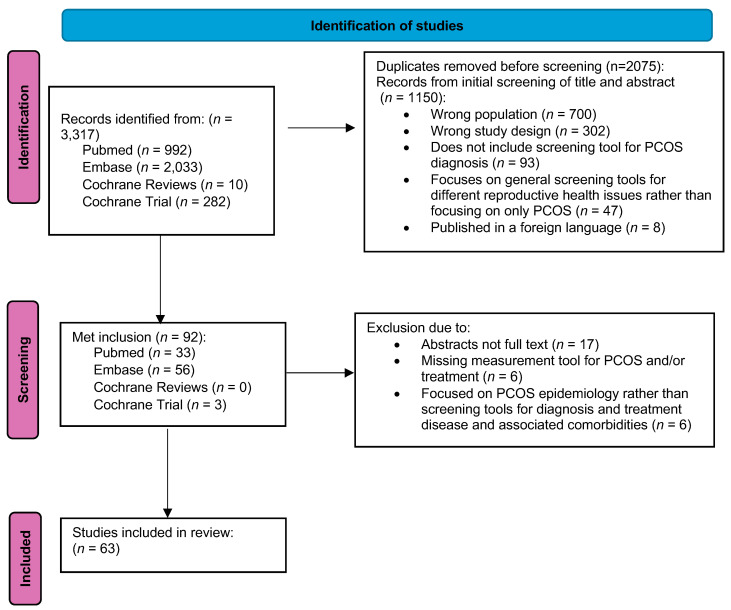
Study selection flow chart.

The initial study extraction resulted in 3317 articles from PubMed (*n* = 992), EMBASE (*n* = 2033), Cochrane Reviews (*n* = 10), and Cochrane Trial (*n* = 282). Following a full-text review, exclusion of duplicates, and exclusion of articles that did not meet our inclusion criteria, a total of 63 studies were retained for analysis (Figure 1).

### 4.1. Major Constructs and SDoH Explored

Of the sixty-three included studies, sixty-one further identified and explored the role of various SDoH on PCOS screening in at-risk women [19,20,21,22,23,24,25,26,27,28,29,30,31,32,33,34,35,36,37,38,39,40,41,42,43,44,45,46,47,48,49,50,51,52,53,54,55,56,57,58,59,60,61,62,63,64,65,66,67,68,69,70,71,72,73,74,75,76,77,78,79,80,81]. Among the thirty-one identified SDoH, those related to patient demographics were most frequently cited (Table S1). Specifically, parameters such as age (*n* = 59), gender (*n* = 10), ethnicity (*n* = 6), race (*n* = 5), nationality (*n* = 1), and sex (*n* = 1) were identified. Family characteristics and support were also frequently highlighted, with specific determinants including marital/partnership status (*n* = 18), family income (*n* = 9), and living conditions/place of residency (*n* = 7), among others. Additionally, employment status, employment benefits, and financial status were recurrent determinants mentioned across included studies, specifically socioeconomic status (*n* = 9), employment (*n* = 15), availability of sick leave (*n* = 1), income level perception (*n* = 1), and insurance (*n* = 1). Other factors include education (*n* = 24), cigarette use (*n* = 5), alcohol use (*n* = 2), religion (*n* = 2), marijuana use (*n* = 1), exercise frequency (*n* = 1), and interests (*n* = 1). Out of the sixty-three studies included, forty-one (66%) recruited participants with a completed PCOS diagnosis status at the start of the study (Table S1).

Included studies characterized the role of sixty-three identified social constructs in PCOS screening in at-risk women. Factors associated with patient demographics and background were often cited, including anthropometric measurements like BMI (*n* = 43), medical history (*n* = 16), demographics (*n* = 8), family history (*n* = 4), family history of psychiatric illness (*n* = 4), medication use (*n* = 3), duration of medication use (*n* = 1), socioeconomic status (*n* = 1), and marital status (*n* = 1). Next, assessment of PCOS-associated conditions and findings were represented, including mental health (*n*= 21), lab findings (*n* = 19), quality of life measurements (*n* = 20), menstrual patterns and irregularities (*n* = 11), measurements of body and facial hair (*n* = 12), presence of PCOS clinical features including acne (*n* = 11), pelvic ultrasound (*n* = 9), infertility (*n* = 8), sexual function/satisfaction (*n* = 5), criteria used for PCOS diagnosis (*n* = 4), insulin sensitivity (*n* = 4), and anovulation (*n* = 1). Finally, less common constructs were noted, including blood pressure (*n* = 4), coping (*n* = 4), PCOS treatment (*n* = 3), body perception (*n* = 3), illness perception (*n* = 2), social functioning (*n* = 2), impact of PCOS diagnosis (*n* = 2), physical functioning and limitations (*n* = 2), bodily pain (*n* = 2), and methods of birth control (*n* = 2), among others (Table S1).

### 4.2. PCOS Screening Tools, Methodology Used, and Measured Associations

A total of 41 tools were validated [19,21,24,30,31,32,33,34,35,36,37,38,39,40,41,42,43,44,45,46,47,49,50,51,53,54,58,59,61,64,65,66,67,68,69,71,72,75,76,77,79,80]. Specific questionnaires that were repeatedly used across studies and translated to different languages included PCOS Quality of Life Questionnaire (PCOSQ-50) (*n* = 14), including a Swedish version (*n* = 1), Chinese version (*n* = 2), and Malay version (*n* = 1); the Short-Form Health Survey (SF-36) (*n* = 8), including a German version (*n* = 1) and version 2 (*n* = 1); World Health Organization Quality of Life questionnaire (WHOQoL-BREF) (*n* = 5); Beck Depression Inventory (*n* = 4), including a Persian version (*n* = 1); General Health Questionnaire (*n* = 2), including versions 30 (*n* = 1) and 28 (*n* = 1); Sexual Quality of Life–Female (SQOL-F) questionnaire (*n* = 2); and the Minnesota Multiphasic Personality Inventory (MMPI) (*n* = 2) (Table S2). The mode of administration included either a single method or multiple methods of data collection such as physical assessment (*n* = 28), in-person and self-administered questionnaires (*n* = 24), personal interviews (*n* = 11), paper-based surveys (*n* = 11), online surveys (*n* = 9), mailed questionnaires (*n* = 5), group discussion (*n* = 1), telephone survey (*n* = 2), and hospital database (*n* = 1). Most frequently reported significant associations were observed between PCOS women and multiple comorbidities including irregular hormonal features/levels (*n* = 26), psychiatric disorders (*n* = 25), demographic and clinical data (*n* = 13), high BMI (*n* = 9), sexual quality of life (*n* = 3), infertility (*n* = 3), body image disturbance and self-esteem (*n* = 2), parity (*n* = 1), and social issues (*n* = 1) (Table S2). Major tool limitations include small sample size (*n* = 18), generalizability (*n* = 15), self-reported bias (*n* = 11), selection bias (*n* = 10), inconsistency of diagnostic criteria, workup, and treatment (*n* = 9), inability to determine causality (*n* = 6), inability to address some concerns in the survey (*n* = 6), and confounding bias (*n* = 5) (Table S2).

### 4.3. Types of Mental Health Disorders and Chronic Pain Associated with PCOS

We reviewed 40 of the included studies which examined the associations between mental health disorders and PCOS [19,21,24,25,26,27,28,29,30,31,32,33,34,35,36,37,38,43,44,45,46,47,50,51,52,53,54,55,56,58,59,61,64,65,66,67,68,69,71,72,75,76] (Table 1). These studies encompassed a diverse array of mental health conditions, including depression, anxiety, psychological distress, psychosis, and bipolar disorder. The instruments utilized to assess these conditions varied widely, incorporating as few as 2 items to as many as 568 items in more comprehensive inventories. A significant association between mental health disorders and PCOS was reported in 34 of these studies, underscoring a substantial link between these conditions. Additionally, our review identified five studies that explored the relationship between chronic pain and PCOS. Measurement tools in these studies ranged from singular items to detailed 36-item surveys. Notably, four of these studies reported significant findings, indicating a prevalent association between chronic pain and PCOS (Table 1).

### 4.4. Validation and Implementation Strategies for PCOS Screening Tools

A total of forty-seven studies described either validation or implementation strategies [19,20,21,22,24,26,27,28,32,35,37,38,39,41,42,43,44,46,47,49,50,51,52,54,57,59,60,61,62,63,64,65,66,67,68,69,70,71,72,73,74,75,76,77,78,79,80,81]. Thirty-one out of sixty-three studies (67%) described the validation methods for their PCOS screening tools. The predominant method (*n* = 13, 21%) involved statistical analysis to confirm the efficacy of the screening tools, with most authors opting to use chi-squared analysis for categorical data or independent *t*-tests for continuous data. To ensure their data were normalized, authors opted to use Shapiro–Wilks analysis or Kolmogorov–Smirnov analysis. Eleven studies (*n* = 9, 14%) benchmarked the screening tools against established criteria, including the Rotterdam criteria (*n* = 3, 5%), the SF-36 (*n* = 3, 5%), and the PCOSQ (*n* = 5, 8%). Two studies (*n* = 2, 3%) utilized clinical parameters such as hair growth, menstrual cyclicity, BMI, and blood pressure to validate the screening tools (Table 2).

Sixteen of sixty-three studies (*n* = 16, 25%) outlined implementation strategies for their PCOS screening tools. The most frequently mentioned strategy was the addition of PCOS screening tools alongside mental health assessments (*n* = 9, 14%) to address the psychopathological dimensions of PCOS, potentially enhancing treatment adherence and doctor–patient interactions. Five studies (*n* = 5, 8%) recommended the integration of PCOS screening during primary care visits to facilitate early symptom detection. One study developed a survey aimed at forming diagnostic criteria and guidelines for PCOS diagnosis. Another study was designed for clinical trial purposes to assess the effectiveness of PCOS treatments (Table 2).

### 4.5. Lessons Learned and Future Recommendations

Using the three phases of qualitative content analysis delineated by Elo and Kyngas [16], qualitative themes were identified. First, data relevant to lessons learned were collected from each of the included studies in the preparation stage (Phase I). Second, lessons learned were organized into bullet points and tabulated by primary author to compare data across studies and explore emerging themes (Phase 2). Major themes are highlighted in Table 3.

### 4.6. Critical Appraisal Skills Programme (CASP) Checklist

All of the studies in this review discussed the study aims and selection methods of the subjects, while 94% (*n* = 59) of the studies described the study design. Valid measures were utilized in 76% (*n* = 48) of the studies. When explaining the statistical analysis of the studies, only 25% (*n* = 16) stated statistical power, and 46% (*n* = 29) stated response rate. Additionally, when providing the results, 86% (*n* = 54) of studies stated statistical significance and 41% (*n* = 26) stated confidence intervals. In discussing results, only 68% (*n* = 43) of the studies mentioned selection bias, and only 60% (*n* = 38) mentioned generalizability. Overall, the quality scores of the studies all ranged from 4 (*n* = 4, 6%) to 10 (*n* = 1, 2%) out of 10. A total of 9% (*n* = 5) of studies had a score of 5, 21% (*n* = 13) of studies had a score of 6, 29% (*n* = 18) of studies had a score of 7, 17% (*n* = 11) had a score of 8, and 16% (*n* = 10) had a score of 9. Studies with a quality score of 6 or 7, 40% of the studies in this review, indicate a quality score of moderate rigor (Table 4).

**Table 1:** Measurement of chronic pain and mental health criteria as part of PCOS screening tools.

Article No.	Primary Author/Year	Measurement of Mental Health Disorders	Type of Mental Health Disorders	Number of Mental Health Survey Items	Measurement of Chronic Pain	Type of Chronic Pain	Number of Chronic Pain Survey Items	Significance of Association
1	Böttcher et al., 2017 [19]	Yes	Depression and anxiety	PCOSQ: emotions (8 items). HADS: anxiety (7 items). HADS: depression (7 items).	No	N/A	N/A	Significant (mental health)
2	Ding et al., 2022 [21]	Yes	Depression	CDI (27 items).	No	N/A	N/A	N/A
3	Guyatt et al., 2004 [24]	Yes	Depression and anxiety	PCOSQ: emotions (8 items).	No	N/A	N/A	N/A
4	Hariprasath et al., 2023 [25]	Yes	Depression, anxiety	Emotions (21 items). Body hair (2 items). Weight (2 items). Menstrual problem (5 items). Social (3 items).	N/A	N/A	N/A	Significant (depression and overall psychological morbidity) Significant (decreased QoL in overall health and sex)
5	Hollinrake et al., 2007 [16]	Yes	Depression, eating disorders	PRIME-MD-PHQ (26 items). BDI: emotional, cognitive, motivational, and physiological (21 items total).	No	N/A	N/A	Significant (mental health—depression) Significant (mental health—eating disorder)
6	Hussain et al., 2015 [27]	Yes	MDD, dysthymia, panic disorder, OCD, suicidality, bipolar, GAD	Psychiatric diagnosis (10 items).	No	N/A	N/A	Significant (comorbid psychiatric illness)
7	Jedel et al., 2008 [28]	Yes	Depression, anxiety	PCOSQ: emotions, body hair, weight concerns, infertility concerns, and menstrual irregularities (26 items total).	No	N/A	N/A	N/A
8	Jedel et al., 2010 [29]	Yes	Depression, anxiety	BSA-S (9 items). MADSR-S (9 items).	Yes	Physical discomfort	1 item from the BSA-S scale	Significant (mental health) Significant (pain)
9	Jones et al., 2004 [30]	Yes	Depression, anxiety	PCOSQ: emotions (8 items). SF-36: physical functioning (10 items), physical role (4 items), pain (2 items), general health (5 items), vitality (4 items), social function (2 items), emotional role (3 items), and mental health (5 items).	No	N/A	N/A	N/A
10	Joshi et al., 2021 [31]	Yes	Depression, anxiety, and body image disturbances and self-esteem	21 (BDI), 17 (HDRS), 14 (HARS), 19 (BICI), 10 (RSES).	No	N/A	N/A	Nonsignificant (no statistically significant correlation of depression was seen with body image or self-esteem) Significant (PCOS and depression and anxiety prevalence)
11	Karjula et al., 2017 [32]	Yes	Depression, psychological distress, and anxiety	HSCL-25—depression (15 items), anxiety (10 items).	No	N/A	N/A	Significant (depression, psychological distress, and anxiety)
12	Karjula et al., 2020 [33]	Yes	Anxiety and depression	HSCL-25—depression (15 items), anxiety (10 items).	No	N/A	N/A	Significant (anxiety and depression)
13	Karjula et al., 2021 [34]	Yes	Psychosis, schizophrenia	SAS (40 items), PAS (61 items).	No	N/A	N/A	Significant (psychosis, schizophrenia)
14	Klipstein et al., 2006 [35]	Yes	Bipolar disorder	MDQ (15 items).	No	N/A	N/A	Significant (bipolar disorder)
15	Kocak et al., 2022 [36]	Yes	Depression, emotional status	BDI (21 items), PCOSQ—emotional status (8 items).	No	N/A	N/A	Significant (depression, emotional status)
16	Kolahi et al., 2015 [37]	No	N/A	N/A.	No	N/A	N/A	N/A
17	Kumarapeli et al., 2010 [38]	Yes	Psychological distress	GHQ30 (30 items).	No	N/A	N/A	Significant (psychological distress)
18	Maleki et al., 2022 [43]	Yes	Anxiety and depression	Depression (7 items) and sleep and anxiety (7 items).	No	N/A	N/A	Significant (anxiety and depression)
19	Mei et al., 2022 [44]	Yes	Emotional problems	Emotions (8 items).	Yes	Body pain	SF-36v2 (2 items)	N/A
20	Mojahed et al., 2023 [45]	Yes	Depression	BDI (21 items).	No	N/A	N/A	Significant (depression)
21	Nasiri-Amiri et al., 2016 [46]	Yes	Psychosocial and emotional, anxieties/concerns	Psychosocial and emotional (31 items), anxieties/concerns (25 items).	No	N/A	N/A	Significant (psychosocial and emotional, anxieties/concerns)
22	Nasiri-Amiri et al., 2018 [47]	Yes	Psychosocial and emotional wellbeing	1 domain—psychological and Emotional.	No	N/A	N/A	Significant (mental and emotional disorders)
23	Ou et al., 2015 [50]	Yes	Emotional wellbeing, psychological health, and anxiety/depression	3 domains—emotion, psychological. health, and anxiety/depression.	No	N/A	N/A	Significant (emotional disturbances)
24	Panico et al., 2017 [51]	Yes	Obsessive–compulsive, depression, anxiety, paranoid ideation, psychoticism, emotional role function, and mental health domains	8 domains—obsessive–compulsive, depression, anxiety, paranoid ideation, psychoticism, emotional role, mental health, and emotion (×2).	No	N/A	N/A	Significant (psychosocial dysfunction)
25	Patil et al., 2022 [52]	Yes	Anxiety and depression	N/A.	No	N/A	N/A	Significant (psychological disturbances)
26	Patten et al., 2023 [53]	Yes	Emotional problems, emotional wellbeing, depression, and anxiety	6 domains—depression, anxiety, stress, emotions (x2), and role limitations due to emotional problems. There were 36 survey items total.	No	N/A	N/A	Significant (depression, anxiety, and stress)
27	Petkova et al., 2018 [54]	Yes	Psychosocial and emotional wellbeing	1 domain—emotion.	No	N/A	N/A	Nonsignificant (emotional responses)
28	Prathap et al., 2018 [55]	Yes	Anxiety and depression	1 domain—psychological.	No	N/A	N/A	Significant (anxiety and depression)
29	Radwan et al., 2023 [56]	Yes	Psychological distress	2 survey items.	No	N/A	N/A	Significant (psychological stress)
30	Robinson et al., 2020 [58]	Yes	ADHD and anxiety	5 domains in the SDQ survey—emotional symptoms, peer-relationship problems, conduct problems, hyperactivity/inattention, and prosocial problems. The VADPRS had 53 survey items.	No	N/A	N/A	Significant (offspring anxiety, behavioral problems and mental disorders at 7 or 8 years in offspring)
31	Rodrigues et al., 2012 [59]	Yes	Anxiety, mood, and somatoform disorders	20 survey items.	No	N/A	N/A	Significant (mental health disorders)
32	Rzo’nca et al., 2018 [61]	Yes	Psychological symptoms	1 WHOQOL-BREF—domain (psychological).	Yes	Bodily pain	1 WHOQOL-BREF—domain (physical health)	Significant (perceived health, quality of life in physical, psychological, environmental, and social domains compared to controls)
33	Santos et al., 2022 [64]	Yes	Depression and anxiety	21 items in DASS-21.	No	N/A	N/A	Significant (anxiety and depression)
34	Sari et al., 2020 [65]	Yes	Major depression, dysthymia, cyclothymia, bipolar disorder, schizoaffective disorders, schizophrenia, panic disorder, separation anxiety disorder, severe anxiety/generalized anxiety disorder, obsessive–compulsive disorder (OCD), attention deficit/hyperactivity disorder (ADHD), conduct disorder, oppositional defiant disorder, enuresis, encopresis, eating disorders, tic disorders, alcohol and substance addiction, and post-traumatic stress disorder (PTSD)	27 items in the Children’s Depression Inventory (CDI) and KSADS-PL (interview question items).	No	N/A	N/A	Significant (psychiatric disorders, especially major depressive disorder) Nonsignificant (anxiety disorder, OCD, and ADHD)
35	Sayyah-Melli et al., 2015 [66]	Yes	Depressive disorders, anxiety disorders, and major psychopathological disorders	72 items in MMPI.	No	N/A	N/A	Significant (anxiety disorder, mood disorder, depressive disorder, personality disorder, schizoaffective disorder)
36	Scaruffi et al., 2014 [67]	Yes	Personality disorders, depression, bipolar disorder types I and II, and anxiety	175 items in MCMI-III.	No	N/A	N/A	Significant (schizoid, depressive, sadistic, negativistic, masochistic, avoidant, dependent, histrionic, narcissistic, and obsessive–compulsive personality disorders; anxiety, somatoform disorder, bipolar disorder, and major depressive disorder, delusional disorder, and thought disorder)
37	Scaruffi et al., 2018 [68]	Yes	Alexithymia, body image disorders, overall mental health issues	20 items, 71 items, 567 items, respectively.	No	N/A	N/A	Significant (alexithymia and greater body uneasiness, depression, hysteria, psychasthenia, and hypomania)
38	Shakil et al., 2020 [69]	Yes	Depression	36 items in SSDS, 5 items in LSS.	No	N/A	N/A	Significant (depression)
39	Shishehgar et al., 2016 [71]	Yes	Mental component summary scale (MCS), using responses from the 8 domains of HRQOL: physical functioning (PF), role limitation due to physical problem (RP), bodily pain (BP), general health perception (GH), vitality (VT), social functioning (SF), role limitation due to emotional problem (RE), and mental health (MH)	36 items in Short-Form Health Survey 36.	Yes	Physical component summary scale (PCS), using responses from the 8 domains of HRQOL: physical functioning (PF), role limitation due to physical problem (RP), bodily pain (BP), general health perception (GH), vitality (VT), social functioning (SF), role limitation due to emotional problem (RE), and mental health (MH)	36 items in Short-Form Health Survey 36	Significant (mental and physical component summary scales)
40	Sidra et al., 2019 [72]	Yes	Depression	12 items in the SF-12 questionnaire to assess QOL scores with reference to depression, anxiety, sexual dysfunction, social problems.	N/A	N/A	N/A	Significant (depression)
41	Varadan et al., 2019 [75]	N/A	N/A	N/A.	Yes	Oxidative stress (periodontitis)	One item as part of the MDA lab test	Significant (chronic pain)
42	Varanasi et al., 2018 [76]	Yes	Depression, anxiety, emotional wellbeing	10 items in the Kessler Psychological Distress Scale (K10) to assess depression and anxiety and 1 psychological domain; mental health.	No	N/A	N/A	Significant (depression)

**Table 2:** Strategies used for validation, dissemination, and implementation of PCOS screening tools.

Article No.	Primary Author/Year	Strategies Used for Validation, Dissemination, and Implementation
**1**	Böttcher et al., 2017 [19]	Construct validity was tested by comparison with SF-36. The proposed factorial structure can be used in clinical practice because the subscales seem to be suited to the assessment of different aspects of the HRQOL in patients with PCOS.
**2**	Ding et al., 2022 [21]	A warning model that can calculate the absolute risk of depression outcomes in adolescents with PCOS would assist practitioners in identifying at-risk patients and subsequently developing prevention and control strategies. This model will utilize a cross-validation method.
**3**	Dou et al., 2016 [22]	WC, BMI, and PBF can all be used to screen and diagnose PCOS. PBF can be used to screen for PCOS in high-risk Chinese women of reproductive age as it is more sensitive, whereas BMI can be used to diagnose PCOS as it is more specific.
**4**	Guyatt et al., 2004 [24]	Construct validity was tested by correlating PCOSQ domain measures with measures of hair growth, menstrual cyclicity, and hyperandrogenemia at baseline and 44 weeks. Longitudinal validity was tested by determining correlations between changes in clinical parameters and changes in the 5 PCOSQ domains. Future investigators can use the PCOSQ to evaluate treatment effectiveness in PCOS clinical trials as it seems to be responsive to changes in HRQOL in PCOS women.
**5**	Hollinrake et al., 2007 [26]	The Primary Care Evaluation of Mental Disorders Patient Health Questionnaire (PRIME-MD PHQ) has been previously validated for use in gynecology outpatients. The PRIME-MD PHQ is useful in a primary care setting to screen, evaluate, and diagnose mental disorders based on DSM-IV criteria.
**6**	Hussain et al., 2015 [27]	Practitioners should be aware of the prevalence of psychiatric disorders and properly screen for them in routine evaluations of PCOS patients.
**7**	Jedel et al., 2008 [28]	The Swedish version of the PCOSQ is reliable and should be used to measure HRQOL in PCOS patients. Test–retest demonstrated reliability for items and domains over a 7-day timeframe.
**8**	Jones et al., 2004 [30]	Construct validity was tested by comparison with the SF-36 and PCOSQ. The PCOSQ was determined to be a reliable method of measuring HRQOL in PCOS patients, but validity would be improved with an acne dimension incorporated.
**9**	Karjula et al., 2017 [32]	Women with PCOS present with increased symptoms of anxiety and depression and coexistence of these morbidities until premenopausal age, thus raising the need for screening for these symptoms in clinical practice.
**10**	Klipstein et al., 2006 [35]	The high sensitivity and specificity observed using the MDQ in this population relative to patients’ self-reported historical diagnoses of bipolar disorder are consistent with previous reports in psychiatric populations and underscore its utility as an initial screening tool for bipolar illness.
**11**	Kolahi et al., 2015 [37]	The reliability and validity of the Carver Coping Questionnaire have been studied in Iran, and the results revealed that this scale is a valid instrument for measuring coping skills.
**12**	Kumarapeli et al., 2010 [38]	WHOQOL-BREF was found to be a valid and reliable tool to assess the HRQoL of women with PCOS with good convergent and discriminant validity. The Goldberg’s GHQ30 is a self-administered questionnaire that has been previously validated and widely used in Sri Lanka.
**13**	Lam et al., 2005 [39]	The 2003 Rotterdam new diagnostic criteria for polycystic ovarian syndrome are generally applicable to the Hong Kong Chinese population.
**14**	Lerchbaum et al., 2013 [41]	HbA1c and FG are not suitable as screening tools for prediabetes in a large cohort of PCOS women but do show a good level of agreement with T2DM. For such women, an oral glucose tolerance test should be performed for screening of prediabetes.
**15**	Lin et al., 2016 [42]	This study validated Chi-PCOSQ in terms of its responsiveness, longitudinal validity, and measurement invariance. Construct validity was confirmed by significant correlation between the domains of Chi-PCOSQ and generic HRQoL measures (WHOQOL-BREF, EQ-5D) and clinical parameters (body mass index, waist—hip ratio, blood pressure).
**16**	Maleki et al., 2022 [43]	Women with PCOS should be routinely screened for sexual quality of life by qualified health professionals.
**17**	Mei et al., 2022 [44]	The Malay version of the Polycystic Ovary Syndrome Questionnaire is a reliable and valid tool for assessing the health-related quality of life among women in the local population. Validity was assessed through convergent and discriminant validity. Examining the correlation between similar content of the Malay version of the Polycystic Ovary Syndrome Questionnaire and the SF-36 assessed the convergent validity. The discriminant validity was assessed using the known group comparison.
**18**	Nasiri-Amiri et al., 2016 [46]	Using both qualitative and quantitative approaches, a specific, reliable, valid and applicable questionnaire for the assessment of quality of life in women with polycystic ovary syndrome (PCOSQ-50) was developed. The validity of the questionnaire was established through assessment and confirmation of content, face, construct (exploratory factor analysis), and criterion (concurrent) validity.
**19**	Nasiri-Amiri et al., 2018 [47]	Low loading items were removed from the questionnaire, leading to the development of the 43-item questionnaire grouped into 6 factors: psychosocial and emotional, self-body image, fertility, sexual function, obesity and menstrual disorder, and hirsutism. This led to an acceptable questionnaire model with internal consistency and reliability that can be used for PCOS screening.
**20**	Ning et al., 2013 [49]	The survey utilized can be accessed and considered by future professional societies looking to draft future guidelines of PCOS diagnosis and identify the most common diagnostic practices.
**21**	Ou et al., 2015 [50]	Translating PCOSQ into the Chinese version, Chi-PCOSQ, to make an available health-related quality of life survey for Chinese speaking countries. Concepts were added to the Chi-PCOSQ to account for items that may affect health-related quality of life: acne, hair loss, and feeling frightened to get diabetes.
**22**	Panico et al., 2017 [51]	To carry out psychological screenings via use of the health-related quality of life measurements besides for routine physical, laboratory, and instrumental examinations.
**23**	Patil et al., 2022 [52]	A PCOS clinic for screening and intervention can help women obtain proper treatment from all concerned specialties under one roof. Psychological screenings should be utilized due to the high rates of psychological disturbances amongst those with PCOS. Screening for metabolic disorders and other comorbidities is important due to the high rates of conditions in patients with PCOS.
**24**	Petkova et al., 2018 [54]	Utilization of PCOSQ to monitor and attend to the psychological health of PCOS patients.
**25**	Rasgon et al., 2005 [57]	Initial assessments of reproductive and metabolic status prior to bipolar disorder treatment is crucial for appropriate interventions and a possible PCOS diagnosis.
**26**	Rodrigues et al., 2012 [59]	Practitioners should inquire about PCOS patients’ mental health and refer them to a mental health professional if necessary. A multidisciplinary team can work together to care for the patient.
**27**	Rodriguez et al., 2020 [60]	Statistical analysis used for validation of results. A total of 40 different articles were used to determine the prevalence for the variables used in the study before those variables were incorporated into the Bayesian network. More data from different sources will be used to train the network to make improved conditional dependencies and make adjustments to the Bayesian network.
**28**	Rzo’nca et al., 2018 [61]	Statistical analysis used for validation of results. WHOQOL-BREF and SWLS questionnaire reliabilities were measured by Cronbach’s α.
**29**	Salva-Pastor et al., 2020 [62]	Statistical analysis used for validation of results. Possibility of sampling errors was mitigated significantly by having a single experienced operator guided by a standardized TE protocol for NAFLD diagnosis using the FibroScan ^®^ 502 Touch, as well as regular machine inspections and validation. Use of Fibroscan over ultrasound provided greater validation.
**30**	Sánchez-Ferrer et al., 2017 [63]	Statistical analysis used for validation of results. AGD (anogenital distance) can be used as a biomarker for PCOS prevention and can be integrated into clinical practice.
**31**	Santos et al., 2022 [64]	Statistical analysis used for validation of results.
**32**	Sari et al., 2020 [65]	Statistical analysis used for validation of results. Turkish validity and reliability study of the CDI was performed by Oy in 1991. Turkish validity and reliability study of the BIS was performed by Hovardaoglu. The validity and reliability study of the schedule in a Turkish sample was performed by Gokler et al.
**33**	Sayyah-Melli et al., 2015 [66]	Statistical analysis used for validation of results. Recommending assessment of mental and social status of PCOS patients prior to starting treatments. Recommending that psychiatric counseling be provided for the treatment and care of PCOS patients.
**34**	Scaruffi et al., 2014 [67]	Statistical analysis used for validation of results. Describes the shortcomings of current diagnostic criteria and suggests the implementation of more rigorous and specific criteria. Highlights that recognizing and addressing psychopathological aspects of PCOS may lead to improvements in doctor–patient interaction, enhancing sensitivity, and potentially increasing adherence to diagnostic and therapeutic interventions.
**35**	Scaruffi et al., 2018 [68]	Statistical analysis used for validation of results. Highlights that recognizing and addressing psychopathological aspects of PCOS may lead to improvements in doctor–patient interaction, enhancing sensitivity and potentially increasing adherence to diagnostic and therapeutic interventions.
**36**	Shakil et al., 2020 [69]	Statistical analysis used for validation of results. Implementing the referral of PCOS patients to psychologists or sexologists for targeted treatments to improve quality of life and sexual functioning.
**37**	Shaman et al., 2017 [70]	Statistical analysis used for validation of results. Implementing screening measures for age, BMI, insulin resistance, and ethnicity in women with PCOS would allow for early intervention and prevention of MS and CVD.
**38**	Shishehgar et al., 2016 [71]	Statistical analysis used for validation of results.
**39**	Sidra et al., 2019 [72]	Since PCOS has pronounced effects on QOL, screening for metabolic disorders and reproductive health is important due to high rates of conditions of patients with diagnosis. Physicians should implement the use of stress management programs to significantly reduce stress and mental health disorders in PCOS patients.
**40**	Smith et al., 2021 [73]	To ensure accurate participant recruitment, subjects were recruited through an online market research company experienced in compound survey sampling. Ability to understand/read English and currently living in Australia to guarantee accurate interpretation of the purpose of the study. A 3 × 2 factorial design used where participants were assigned to one of six hypothetical scenarios. Study piloted with sample recruited through social media to test the feasibility of questionnaire and estimate effective sizes needed to calculate the required sample size. Participants invited to explain answers to primary outcomes and reminded to answer as described scenario occurred. Even sample size of 100 participants per group (total = 600) to guarantee adequate power and detect significant statistical differences.
**41**	Talpur et al., 2023 [74]	Consecutive sampling of female subjects with no probability was used. Study carried out in the related-specialty department. Effect modifiers such as obesity and hypertension were stratified to examine how they affect PCOS.
**42**	Varadan et al., 2019 [75]	Patients satisfying both inclusion/exclusion criteria were included until a sample size of 30 PCOS and 30 healthy patients was determined to avoid selection bias. In total, 30 participants were designated to each group to allow for a more valid and reliable comparison. In total, 30 PCOS based on Rotterdam criteria and 30 healthy patients were selected for a periodontal examination and a standard oral hygiene care by a specialist who was blinded to both control groups.
**43**	Varanasi et al., 2018 [76]	A 2-item questionnaire to women reporting PCOS diagnosis focusing on reproductive health and mental health based on components of PCOS-Q. A 1-item questionnaire focusing on reproductive health questions to women not reporting PCOS in order to compare differences in comorbidities between both groups. Providers should monitor mental health of PCOS patients due to their high rates of psychological disturbances. Utilization of criteria with set parameters to avoid over diagnosing PCOS and the development of mental health disorders.
**44**	Vutyavanich et al., 2007 [77]	Blood collection was taken in the morning for accurate results of hormonal tests to ensure a more accurate diagnosis of PCOS. Large sample size to estimate and guarantee a 95% confidence interval for prevalence of PCOS.
**45**	Wang et al., 2023 [78]	In total, 285 PCOS patients using Rotterdam inclusion criteria and 201 healthy women were screened to assessed tongue and pulse diagnosis as noninvasive detection methods of PCOS syndrome used in traditional Chinese medicine. Machine learning classification algorithms were used to establish PCOS risk prediction with adequate accuracy.
**46**	Yan et al., 2021 [79]	A questionnaire to evaluate which PCOS diagnostic criteria are mostly used across China was prepared by four reproductive endocrinology specialists and filled out by 30 obstetricians and gynecologists before submitting it to specialists throughout the largest online continuing education platform for further evaluation.
**47**	Zhang et al., 2012 [80]	Practitioners carried out medical history and laboratory tests to account for issues that may affect PCOS. Utilization of Rotterdam diagnostic criteria for PCOS is generally more applicable in Chinese population because hyperandrogenism is present only in half of the patients who participated in the study.

**Table 3:** Lessons learned and future directions.

Major Themes in Lessons Learned
The ideal PCOS screening tool can identify disease-specific effects on QoL such as overweight, acne, hirsutism, infertility, and mental illness to help identify ways to improve QoL in PCOS patients. Metabolic parameters can help identify patients with PCOS, though there is no standard reference using metabolic parameters. Need to identify and treat PCOS patients at risk for or diagnosed with depression and other psychiatric disturbances, which can help decrease prevalence. Protein markers can identify PCOS patients. Need for culturally relevant educational material to reduce stigma and discrimination against and to increase self-esteem among women with PCOS and to increase overall awareness of PCOS. Further studies should focus on how race and ethnic differences influence the clinical presentation, phenotypes, and diagnostic criteria. Limited exposure to patients with PCOS and time during patient visits limits effective identification of problems and care for PCOS patients. The PCOSQ should be tested on large populations to increase its statistical power and applicability to diverse populations. Lifestyle interventions for PCOS patients should include diet and physical activity, and clinical trials may help support this method. The effect of diet on PCOS symptomology warrants study. The impact of maternal PCOS on offspring, including anxiety and hirsutism, warrants further research with emphasis on the role of maternal androgens and inflammatory cytokines in pregnancy. Further studies should focus on the etiology of PCOS, diagnostic tools, and the impact of diagnosis on QoL while prioritizing larger sample sizes. PCOS-associated clinical conditions and complications are a priority when selecting holistic approaches to treatment, with exercise and diet being of great importance. Lower QoL among PCOS patients is associated with sexual dysfunction. Future studies should focus on stratifying the risk of cardiovascular events associated with a PCOS diagnosis.

**Table 4:** CASP checklist.

Study No.	Study Aim(s)	Study Design	Selection of Subjects	Selection Bias	Sample Generalizability	Stat Power	Response Rate	Valid Measures	Stat Sig	CI	Quality Score
*1*	Yes	Yes	Yes	Yes	Yes	Yes	Yes	Yes	Yes	No	9
*2*	Yes	Yes	Yes	No	No	No	Yes	No	Yes	No	5
*3*	Yes	Yes	Yes	Yes	Yes	No	No	Yes	No	No	6
*4*	Yes	Yes	Yes	Yes	Yes	No	No	Yes	No	No	6
*5*	Yes	Yes	Yes	Yes	Yes	No	No	Yes	Yes	Yes	8
*6*	Yes	Yes	Yes	No	Unsure	No	Yes	Unsure	Yes	Yes	6
*7*	Yes	Yes	Yes	No	Unsure	No	Yes	Yes	Yes	Yes	7
*8*	Yes	Yes	Yes	No	No	No	No	Yes	No	No	4
*9*	Yes	Yes	Yes	No	Unsure	No	Yes	Yes	Yes	Yes	7
*10*	Yes	Yes	Yes	No	Yes	No	Yes	No	No	No	5
*11*	Yes	Yes	Yes	No	No	No	Yes	Yes	No	No	5
*12*	Yes	Yes	Yes	Yes	Yes	Unsure	Yes	Yes	Yes	Yes	9
*13*	Yes	Yes	Yes	Yes	Yes	Yes	Yes	Yes	Yes	Yes	10
*14*	Yes	Yes	Yes	Yes	No	Yes	No	Yes	Yes	No	7
*15*	Yes	Yes	Yes	No	Unsure	No	Yes	Yes	Yes	Yes	7
*16*	Yes	Yes	Yes	Yes	Yes	No	Yes	Yes	Yes	Yes	9
*17*	Yes	Yes	Yes	Yes	Yes	No	No	Yes	Yes	Yes	8
*18*	Yes	Yes	Yes	Yes	No	No	Yes	Yes	Yes	Yes	8
*19*	Yes	Yes	Yes	Yes	Yes	Yes	No	Yes	Yes	Yes	9
*20*	Yes	Yes	Yes	No	No	No	No	Yes	Yes	Yes	6
*21*	Yes	Yes	Yes	No	No	No	Yes	Yes	Yes	Yes	7
*22*	Yes	Yes	Yes	Yes	Yes	No	Unsure	Yes	Yes	No	7
*23*	Yes	Yes	Yes	Yes	Yes	No	No	No	No	No	5
*24*	Yes	Yes	Yes	Yes	Yes	No	No	No	Yes	No	6
*25*	Yes	Yes	Yes	Yes	Yes	No	Yes	Yes	Yes	Yes	9
*26*	Yes	Yes	Yes	Yes	No	Yes	Yes	Yes	Yes	Yes	9
*27*	Yes	Yes	Yes	Yes	Yes	No	Yes	Yes	Yes	Yes	9
*28*	Yes	Yes	Yes	Yes	No	No	No	Yes	Yes	Yes	7
*29*	Yes	Yes	Yes	Yes	Yes	No	No	Yes	Yes	Yes	8
*30*	Yes	Yes	Yes	Yes	No	Yes	No	Yes	Yes	No	8
*31*	Yes	Yes	Yes	Yes	No	No	Yes	No	Yes	Yes	7
*32*	Yes	Yes	Yes	Yes	No	No	Yes	No	Yes	No	6
*33*	Yes	Yes	Yes	Yes	Yes	No	No	Yes	Yes	No	7
*34*	Yes	No	Yes	No	No	No	No	Yes	Yes	No	4
*35*	Yes	Yes	Yes	Yes	No	No	No	No	No	No	4
*36*	Yes	Yes	Yes	Yes	Yes	Yes	Yes	Yes	Yes	No	9
*37*	Yes	Yes	Yes	Yes	Yes	No	Yes	Yes	No	No	7
*38*	Yes	Yes	Yes	Yes	Yes	No	No	Yes	Yes	No	7
*39*	Yes	Yes	Yes	Yes	Yes	Yes	No	Yes	Yes	No	8
*40*	Yes	Yes	Yes	Yes	Yes	Yes	Yes	No	Yes	No	8
*41*	Yes	Yes	Yes	Yes	Yes	No	Yes	Yes	No	Yes	8
*42*	Yes	Yes	Yes	No	No	Yes	No	Yes	Yes	No	6
*43*	Yes	Yes	Yes	No	Yes	No	No	Yes	Yes	No	6
*44*	Yes	Yes	Yes	No	No	Yes	Yes	Yes	Yes	No	7
*45*	Yes	Yes	Yes	Yes	Yes	No	No	No	Yes	Yes	7
*46*	Yes	Yes	Yes	Yes	Yes	No	No	Yes	Yes	Yes	8
*47*	Yes	Yes	Yes	Yes	No	No	Yes	Yes	Yes	Yes	8
*48*	Yes	Yes	Yes	No	Yes	No	No	Yes	Yes	No	6
*49*	Yes	Yes	Yes	Yes	Yes	No	No	Yes	Yes	No	7
*50*	Yes	No	Yes	Yes	Yes	No	No	Yes	Yes	No	6
*51*	Yes	Yes	Yes	No	Yes	No	No	Yes	Yes	No	6
*52*	Yes	Yes	Yes	Yes	Yes	No	No	Yes	Yes	No	7
*53*	Yes	Yes	Yes	No	No	No	No	No	Yes	No	4
*54*	Yes	Yes	Yes	Yes	Yes	Yes	Yes	Yes	Yes	No	9
*55*	Yes	Yes	Yes	No	No	Yes	Yes	Yes	Yes	No	7
*56*	Yes	Yes	Yes	Yes	Yes	Yes	No	Yes	Yes	Yes	9
*57*	Yes	Yes	Yes	No	Yes	No	No	No	Yes	No	5
*58*	Yes	No	Yes	Yes	No	Yes	No	Yes	Yes	No	6
*59*	Yes	Yes	Yes	No	Yes	Yes	Yes	Yes	Yes	No	7
*60*	Yes	No	Yes	Yes	Yes	No	No	No	Yes	Yes	6
*61*	Yes	Yes	Yes	Yes	No	No	No	No	Yes	No	5
*62*	Yes	Yes	Yes	Yes	Yes	No	Yes	No	Yes	Yes	8
*63*	Yes	Yes	Yes	Yes	Yes	No	Yes	Yes	Yes	No	7

## 5. Discussion

Patients with PCOS are at an increased risk of mental health conditions, such as depression and anxiety, compared to those without the condition [5,6,7]. These findings are being reported globally, and the reasons behind this increased prevalence are multicomplex, whether physiological, psychological, or social factors, all of which have detrimental effects on the quality of life of patients with PCOS [82,83,84]. However, the association between PCOS and mental health is often overlooked by healthcare providers, primarily due to a lack of awareness [85,86]. Similarly, chronic pain related to PCOS is frequently under-recognized and inadequately addressed [87,88]. Martin et al. revealed that the most common concern among individuals with PCOS is pain- and discomfort-related symptoms; however, validated PCOS questionnaires or instruments lack the integration of symptoms within the measures [87]. For instance, PCOSQ only incorporates one pain-related question, headache, leaving out all other pain-related symptoms, like menstrual cramping, bleeding, and bloating, while the updated version, PCOSQ-50, includes no pain item questions at all [46,87]. It is essential to begin incorporating assessments for mental health and chronic pain into the screening protocols for diagnosing and managing PCOS to foster a comprehensive and holistic approach that aims to enhance the quality of life of patients with PCOS [88,89,90,91,92,93]. Including these assessments will allow providers to gain awareness of and understand the problems experienced by those with PCOS, as well as enable the implementation of strategies for treatment and management [88].

The social determinants of health play a role in exacerbating PCOS symptoms and associated comorbidities, particularly when it comes to worsening mental health and quality of life [1]. Factors such as age, marital/partnership status, socioeconomic status and employment, and education are the most notable determinants of health encountered in this study that often synergize to create poor health outcomes in patients with PCOS. Promoting health literacy among patients with PCOS has proven beneficial, with higher health literacy associated with better health behaviors [94]. Additionally, a recent systematic review by Mun Lau et al. investigating the needs of patients with PCOS suggests that patients themselves seek culturally relevant information related to their condition [94] This creates a large window of opportunity to reach patients to help them meet their health goals [94]. As such, culturally relevant educational programs targeting both patients with PCOS and health practitioners could improve care outcomes, reduce stigma, and enhance the overall quality of life for women with PCOS [94,95,96].

Establishing a diagnosis of PCOS can be challenging and often involves multiple healthcare professionals and significant time. Gibson et al. emphasized that most patients globally often require three or more healthcare professionals, and it takes up to a year until a diagnosis is provided, leading to frustration [90]. Delays in diagnosis can be attributed to the absence of a single diagnostic test, varying diagnostic criteria, lack of clarification within the criteria, and the need for exclusion of other conditions [90]. These challenges are exacerbated by gaps in knowledge and awareness among healthcare providers, leading to an inability to recognize symptoms of PCOS and provide such screening tools even when available [85]. These limitations further emphasize the gap in the availability of validated PCOS screening tools and standardized measures for diagnosis and treatment [90]. There is a need to test for the efficacy of the current screening tools for PCOS [90,91]. Current strategies should reinforce the integration of mental health assessments, as described above, to improve not only the physiological burden experienced by those with PCOS but also the psychological symptoms of anxiety and depression [88,89]. Incorporating screening tools within the first primary medical visit as part of the standard evaluation of women’s health will promote awareness and identification of PCOS promptly and potentially have cost-effective results [92,93]. Once screening is positive, providers can follow the “2023 International Evidence-based Guideline for the Assessment and Management of Polycystic Ovary Syndrome”, updated with more evidence-based diagnostic criteria to help providers efficiently manage PCOS and address patient priorities [91].

The lessons learned from the current body of literature underscore the importance of an integrated, multifaceted approach to PCOS diagnosis and management. This approach can significantly improve the overall quality of life of these individuals by addressing both physical and mental health aspects simultaneously [82,88,89]. The development of holistic screening tools that encompass a wide range of PCOS-related symptoms, including physical manifestations (e.g., overweight, acne, hirsutism, and infertility) and mental health issues, is crucial [82,88,89]. These tools should aim to provide a comprehensive assessment of the patient’s quality of life and specific challenges associated with PCOS [83,93]. Further, there is a pressing need for culturally relevant educational resources that can help reduce the stigma and discrimination faced by PCOS patients. Such materials would not only boost self-esteem among women with PCOS but also raise awareness about the condition, its symptoms, and its comorbidities within various communities [94]. Future research should delve deeper into understanding the etiology of PCOS and developing more effective diagnostic tools [91,92,93]. Emphasis should be placed on studies with larger sample sizes to ensure the findings are robust and applicable to a broader patient population [95]. Additionally, given the positive impact of lifestyle changes on PCOS management, future research should continue to explore diet and physical activity interventions [96,97]. Clinical trials are essential to provide evidence supporting these lifestyle modifications as viable treatment options [96,97]. Finally, infertility is a significant concern for many PCOS patients, and it has been identified as one of the most distressing aspects of the condition [9]. Future tools and research should prioritize addressing infertility, providing patients with comprehensive support and treatment options [97,98]. By incorporating these future directions, healthcare providers can improve the screening process and overall care for women with PCOS, ultimately enhancing their quality of life [94,95,96,97].

### Limitations

Study findings should be analyzed in the context of existing limitations. First, the gray literature and reference list tracing were not included, which may have omitted relevant unpublished studies, case reports, and press releases. Second, only English-language publications were considered for inclusion, excluding potentially pertinent non-English research despite this review’s international scope. Third, some relevant search terms around PCOS, chronic pain, mental health, or screening tools may have been unintentionally discarded given the emergence of new terminology in a rapidly evolving field, particularly pertaining to the standardization of such screening tools. However, the detailed mesh terms, PRISMA guidelines, and protocol support likely minimized such risks. Further validation is needed for existing measurement tools, as our formal quality assessment using the CASP checklist indicated. Future research studies could inform standardization around adequately and comprehensively assessing patient individual, mental, and physical factors influencing the detection of a PCOS diagnosis.

## 6. Conclusions

Our systematic review provides timely and robust initial insights into the current limitations of PCOS screening tools, particularly when it comes to integrating mental health and chronic pain symptoms affecting the overall progression and manifestation of this chronic disease. Results suggest that effective strategies for disseminating and promoting information on PCOS screening successfully should consider SDoH, validation of tools to be culturally relevant, and individualized symptoms of women with PCOS for early detection and improved management of the syndrome.

## Supplementary Materials

The following supporting information can be downloaded at: https://www.mdpi.com/article/10.3390/clinpract14050131/s1, Table S1: Study characteristics; Table S2: Type of methodology used and limitations of measurement tools.

## References

[1] Deswal R., Narwal V., Dang A., Pundir C.S. (2020). The prevalence of polycystic ovary syndrome: A brief systematic review. J. Hum. Reprod. Sci..

[2] Boyle J., Teede H.J. (2012). Polycystic ovary syndrome—An update. Aust. Fam. Physician.

[3] Brady C., Mousa S.S., Mousa S.A. (2009). Polycystic ovary syndrome and its impact on women’s quality of life: More than just an endocrine disorder. Drug Healthc. Patient Saf..

[4] Tabassum F., Jyoti C., Sinha H.H., Dhar K., Akhtar M.S. (2021). Impact of polycystic ovary syndrome on quality of life of women in correlation to age, basal metabolic index, education and marriage. PLoS ONE.

[5] Teede H., Deeks A., Moran L. (2010). Polycystic ovary syndrome: A complex condition with psychological, reproductive and metabolic manifestations that impacts on health across the lifespan. BMC Med..

[6] Brutocao C., Zaiem F., Alsawas M., Morrow A.S., Murad M.H., Javed A. (2018). Psychiatric disorders in women with polycystic ovary syndrome: A systematic review and meta-analysis. Endocrine.

[7] Cooney L.G., Lee I., Sammel M.D., Dokras A. (2017). High prevalence of moderate and severe depressive and anxiety symptoms in polycystic ovary syndrome: A systematic review and meta-analysis. Hum. Reprod..

[8] Dewani D., Karwade P., Mahajan K.S. (2023). The invisible struggle: The psychosocial aspects of polycystic ovary syndrome. Cureus.

[9] Okamura Y., Saito F., Takaishi K., Motohara T., Honda R., Ohba T., Katabuchi H. (2017). Polycystic ovary syndrome: Early diagnosis and intervention are necessary for fertility preservation in young women with endometrial cancer under 35 years of age. Reprod. Med. Biol..

[10] Knowler W.C., Barrett-Connor E., Fowler S.E., Hamman R.F., Lachin J.M., Walker E.A., Nathan D.M., Diabetes Prevention Program Research Group (2002). Reduction in the incidence of type 2 diabetes with lifestyle intervention or metformin. N. Engl. J. Med..

[11] Joham A.E., Piltonen T., Lujan M.E., Kiconco S., Tay C.T. (2022). Challenges in diagnosis and understanding of natural history of polycystic ovary syndrome. Clin. Endocrinol..

[12] Azziz R. (2004). PCOS: A diagnostic challenge. Reprod. Biomed. Online.

[13] Agapova S.E., Cameo T., Sopher A.B., Oberfield S.E. (2014). Diagnosis and challenges of polycystic ovary syndrome in adolescence. Semin. Reprod. Med..

[14] Hoeger K.M., Dokras A., Piltonen T. (2021). Update on PCOS: Consequences, challenges, and guiding treatment. J. Clin. Endocrinol. Metab..

[15] Arksey H., O’Malley L. (2005). Scoping studies: Towards a methodological framework. Int. J. Soc. Res. Methodol..

[16] Buccheri R.K., Sharifi C. (2017). Critical Appraisal Tools and Reporting Guidelines for Evidence-Based Practice. Worldviews Evid Based Nurs..

[17] CASP Checklists—How to Use Them & Why They Are Important. CASP-Critical Appraisal Skills Programme. https://casp-uk.net/how-to-use-checklist/.

[18] Harrison J.K., Reid J., Quinn T.J., Shenkin S.D. (2017). Using quality assessment tools to critically appraise ageing research: A guide for clinicians. Age Ageing.

[19] Böttcher B., Fessler S., Friedl F., Toth B., Walter M.H., Wildt L., Riedl D. (2018). Health-related quality of life in patients with polycystic ovary syndrome: Validation of the German PCOSQ-G. Arch. Gynecol. Obstet..

[20] Conway G., Dewailly D., Diamanti-Kandarakis E., Escobar-Morreale H.F., Franks S., Gambineri A., Kelestimur F., Macut D., Micic D., Pasquali R. (2014). European survey of diagnosis and management of the polycystic ovary syndrome: Results of the ESE PCOS Special Interest Group’s Questionnaire. Eur. J. Endocrinol..

[21] Ding R., Zhou H., Yan X., Liu Y., Guo Y., Tan H., Wang X., Wang Y., Wang L. (2022). Development and validation of a prediction model for depression in adolescents with polycystic ovary syndrome: A study protocol. Front. Psychiatry.

[22] Dou P., Ju H., Shang J., Li X., Xue Q., Xu Y., Guo X. (2016). Application of receiver operating characteristic curve in the assessment of the value of body mass index, waist circumference and percentage of body fat in the Diagnosis of Polycystic Ovary Syndrome in childbearing women. J. Ovarian Res..

[23] Duman G., Sariakcali B., Erşan S., Bakır S. (2022). The impact of Dicer, Drosha, and Exportin-5 levels in polycystic ovary syndrome (PCOS) diagnosis and phenotyping. Endokrynol. Pol..

[24] Guyatt G., Weaver B., Cronin L., Dooley J.A., Azziz R. (2004). Health-related quality of life in women with polycystic ovary syndrome, a self-administered questionnaire, was validated. J. Clin. Epidemiol..

[25] Hariprasath L., Selvakumar D., Dharani V., Durgalakshmi K.K., Abilash V.G., Gopenath T.S., Nishu S. (2023). Infertility and Social Issue Have the Most Significant Impact on Health- Related Quality of Life among Polycystic Ovarian Syndrome Women in South India. J. Hum. Reprod. Sci..

[26] Hollinrake E., Abreu A., Maifeld M., Van Voorhis B.J., Dokras A. (2007). Increased risk of depressive disorders in women with polycystic ovary syndrome. Fertil. Steril..

[27] Hussain A., Chandel R.K., Ganie M.A., Dar M.A., Rather Y.H., Wani Z.A., Shiekh J.A., Shah M.S. (2015). Prevalence of psychiatric disorders in patients with a diagnosis of polycystic ovary syndrome in kashmir. Indian J. Psychol. Med..

[28] Jedel E., Kowalski J., Stener-Victorin E. (2008). Assessment of health-related quality of life: Swedish version of polycystic ovary syndrome questionnaire. Acta Obstet. Gynecol. Scand..

[29] Jedel E., Waern M., Gustafson D., Landen M., Eriksson E., Holm G., Nilsson L., Lind A.K., Janson P.O., Stener-Victorin E. (2010). Anxiety and depression symptoms in women with polycystic ovary syndrome compared with controls matched for body mass index. Hum. Reprod..

[30] Jones G.L., Benes K., Clark T.L., Denham R., Holder M.G., Haynes T.J., Mulgrew N.C., Shepherd K.E., Wilkinson V.H., Singh M. (2004). The polycystic ovary syndrome health-related quality of life questionnaire (PCOSQ): A validation. Hum. Reprod..

[31] Joshi R.D., Sawant N., Mayadeo N.M. (2022). How common are depressive-anxiety states, body image concerns and low self-esteem in patients of PCOS?. J. Obstet. Gynaecol. India.

[32] Karjula S., Morin-Papunen L., Auvinen J., Ruokonen A., Puukka K., Franks S., Järvelin M.R., Tapanainen J.S., Jokelainen J., Miettunen J. (2017). Psychological distress is more prevalent in fertile age and premenopausal women with PCOS symptoms: 15-year follow-up. J. Clin. Endocrinol. Metab..

[33] Karjula S., Morin-Papunen L., Franks S., Auvinen J., Järvelin M.R., Tapanainen J.S., Jokelainen J., Miettunen J., Piltonen T.T. (2020). Population-based data at ages 31 and 46 show decreased HRQoL and life satisfaction in women with PCOS symptoms. J. Clin. Endocrinol. Metab..

[34] Karjula S., Arffman R.K., Morin-Papunen L., Franks S., Järvelin M.R., Tapanainen J.S., Miettunen J., Piltonen T.T. (2022). A population-based follow-up study shows high psychosis risk in women with PCOS. Arch. Womens Ment. Health.

[35] Klipstein K.G., Goldberg J.F. (2006). Screening for bipolar disorder in women with polycystic ovary syndrome: A pilot study. J. Affect. Disord..

[36] Kocak D.Y., Ugurlu M. (2022). Depression symptoms and quality of life in women with polycystic ovary syndrome. Perspect. Psychiatr. Care.

[37] Kolahi L., Asemi N., Mirzaei M., Adibi N., Beiraghdar M., Mehr A.M. (2015). The relationship between quality of life and coping strategies in polycystic ovary syndrome patients. Adv. Biomed. Res..

[38] Kumarapeli V., Seneviratne R.d.A., Wijeyaratne C. (2011). Health-related quality of life and psychological distress in polycystic ovary syndrome: A hidden facet in South Asian women: HRQoL and psychological distress in South Asian women with PCOS. BJOG.

[39] Lam P.M., Ma R.C.W., Cheung L.P., Chow C.C., Chan J.C.N., Haines C.J. (2005). Polycystic ovarian syndrome in Hong Kong Chinese women: Patient characteristics and diagnostic criteria. Hong Kong Med. J..

[40] Lee I.T.L., Sansone S., Irfan M., Copp T., Beidas R., Dokras A. (2022). Implementation of international guidelines for polycystic ovary syndrome: Barriers and facilitators among gynecologists and primary care providers. F&S Rep..

[41] Lerchbaum E., Schwetz V., Giuliani A., Obermayer-Pietsch B. (2013). Assessment of glucose metabolism in polycystic ovary syndrome: HbA1c or fasting glucose compared with the oral glucose tolerance test as a screening method. Hum. Reprod..

[42] Lin C.Y., Ou H.T., Wu M.H., Chen P.C. (2016). Validation of Chinese version of Polycystic Ovary Syndrome Health-related quality of Life Questionnaire (Chi-PCOSQ). PLoS ONE.

[43] Maleki A., Jenabi E., Fereidooni B., Abdoli S. (2023). Predictive factors of Sexual Quality of Life in women with polycystic ovary syndrome: A path analysis. Int. J. Impot. Res..

[44] Mei L.L., Abu M.A., Chew K.T., Ismail A., Zainuddin A.A., Nur Azurah A.G. (2022). The reliability and validity of the Malay version of polycystic ovarian syndrome health-related quality of life questionnaire. Front. Endocrinol..

[45] Mojahed B.S., Ghajarzadeh M., Khammar R., Shahraki Z. (2023). Depression, sexual function and sexual quality of life in women with polycystic ovary syndrome (PCOS) and healthy subjects. J. Ovarian Res..

[46] Nasiri-Amiri F., Ramezani Tehrani F., Simbar M., Montazeri A., Mohammadpour R.A. (2016). Health-related quality of life questionnaire for polycystic ovary syndrome (PCOSQ-50): Development and psychometric properties. Qual. Life Res..

[47] Nasiri-Amiri F., Ramezani Tehrani F., Simbar M., Montazeri A., Mohammadpour R.A. (2018). The polycystic ovary syndrome health-related quality-of-life questionnaire: Confirmatory factor analysis. Int. J. Endocrinol. Metab..

[48] Neubronner S.A., Indran I.R., Chan Y.H., Thu A.W.P., Yong E.L. (2021). Effect of body mass index (BMI) on phenotypic features of polycystic ovary syndrome (PCOS) in Singapore women: A prospective cross-sectional study. BMC Womens Health.

[49] Ning N., Balen A., Brezina P.R., Leong M., Shoham Z., Wallach E.E., Zhao Y. (2013). How to recognize PCOS: Results of a web-based survey at Ivf-worldwide.com. Reprod. Biomed. Online.

[50] Ou H.T., Wu M.H., Lin C.Y., Chen P.C. (2015). Development of Chinese version of Polycystic Ovary Syndrome Health-related quality of Life Questionnaire (Chi-PCOSQ). PLoS ONE.

[51] Panico A., Messina G., Lupoli G.A., Lupoli R., Cacciapuoti M., Moscatelli F., Esposito T., Villano I., Valenzano A., Monda V. (2017). Quality of life in overweight (obese) and normal-weight women with polycystic ovary syndrome. Patient Prefer. Adherence.

[52] Patil A.D., Vaidya R.A., Begum S., Chauhan S.L., Mukherjee S., Kokate P.P., Joshi B.N. (2022). An integrated multidisciplinary model of care for addressing comorbidities beyond reproductive health among women with polycystic ovary syndrome in India. Indian J. Med. Res..

[53] Patten R.K., McIlvenna L.C., Moreno-Asso A., Hiam D., Stepto N.K., Rosenbaum S., Parker A.G. (2023). Efficacy of high-intensity interval training for improving mental health and health-related quality of life in women with polycystic ovary syndrome. Sci. Rep..

[54] Petkova V., Kamusheva M., Manova M., Savova A., Andreevska K. (2018). Polycystic ovary syndrome impact on women’s quality of life: Pilot study. Biomed. Res..

[55] Prathap A., Subhalakshmi T.P., Varghese P.J. (2018). A cross-sectional study on the proportion of anxiety and depression and determinants of quality of life in polycystic ovarian disease. Indian J. Psychol. Med..

[56] Radwan A., Al-Juhani A.A., Alshehri A.A., Alsumaili A.A., Aseri S.K., Alzahrani M.J., Qahwaji D.M., Zaafarani F. (2023). The association of polycystic ovarian syndrome among reproductive-aged women with consumption of junk food in Jeddah, Saudi Arabia. Cureus.

[57] Rasgon N.L., Altshuler L.L., Fairbanks L., Elman S., Bitran J., Labarca R., Saad M., Kupka R., Nolen W.A., Frye M.A. (2005). Reproductive function and risk for PCOS in women treated for bipolar disorder. Bipolar Disord..

[58] Robinson S.L., Ghassabian A., Sundaram R., Trinh M.H., Bell E.M., Mendola P., Yeung E.H. (2020). The associations of maternal polycystic ovary syndrome and hirsutism with behavioral problems in offspring. Fertil. Steril..

[59] Rodrigues C.E.G., Ferreira L.d.L., Jansen K., Lopez M.R.A., Drews Júnior C.R., Souza L.D.d.M. (2012). Evaluation of common mental disorders in women with polycystic ovary syndrome and its relationship with body mass index. Rev. Bras. Ginecol. Obstet..

[60] Rodriguez E.M., Thomas D., Druet A., Vlajic-Wheeler M., Lane K.J., Mahalingaiah S. (2020). Identifying women at risk for Polycystic ovary syndrome using a mobile health app: Virtual tool functionality assessment. JMIR Form. Res..

[61] Rzońca E., Bień A., Wdowiak A., Szymański R., Iwanowicz-Palus G. (2018). Determinants of quality of life and satisfaction with life in women with polycystic ovary syndrome. Int. J. Environ. Res. Public Health.

[62] Salva-Pastor N., López-Sánchez G.N., Chávez-Tapia N.C., Audifred-Salomón J.R., Niebla-Cárdenas D., Topete-Estrada R., Pereznuñez-Zamora H., Vidaltamayo-Ramírez R., Báez-Arellano M.E., Uribe M. (2020). Polycystic ovary syndrome with feasible equivalence to overweight as a risk factor for non-alcoholic fatty liver disease development and severity in Mexican population. Ann. Hepatol..

[63] Sánchez-Ferrer M.L., Mendiola J., Hernández-Peñalver A.I., Corbalán-Biyang S., Carmona-Barnosi A., Prieto-Sánchez M.T., Nieto A., Torres-Cantero A.M. (2017). Presence of polycystic ovary syndrome is associated with longer anogenital distance in adult Mediterranean women. Hum. Reprod..

[64] Santos I.K., Pichini G.S., Daniel D Ferreira C., Dantas P.B., Browne R.A., de Queiros V., Soares G.M., Gonçalves A.K., Cabral B.G., Maranhão T.M.O. (2022). Effects of high-intensity interval training in combination with detraining on mental health in women with polycystic ovary syndrome: A randomized controlled trial. Front. Physiol..

[65] Sari S.A., Celik N., Uzun Cicek A. (2020). Body perception, self-esteem, and comorbid psychiatric disorders in adolescents diagnosed with polycystic ovary syndrome. J. Pediatr. Adolesc. Gynecol..

[66] Sayyah-Melli M., Alizadeh M., Pourafkary N., Ouladsahebmadarek E., Jafari-Shobeiri M., Abbassi J., alsadat Kazemi-Shishvan M., Sedaghat K. (2015). Psychosocial factors associated with polycystic ovary syndrome: A case control study. J. Caring Sci..

[67] Scaruffi E., Gambineri A., Cattaneo S., Turra J., Vettor R., Mioni R. (2014). Personality and psychiatric disorders in women affected by polycystic ovary syndrome. Front. Endocrinol.

[68] Scaruffi E., Franzoi I.G., Civilotti C., Guglielmucci F., La Marca L., Tomelini M., Veglia F., Granieri A. (2019). Body image, personality profiles and alexithymia in patients with polycystic ovary syndrome (PCOS). J. Psychosom. Obstet. Gynaecol..

[69] Shakil M., Ashraf F., Wajid A. (2020). Sexual functioning as predictor of depressive symptoms and life satisfaction in females with Polycystic Ovary Syndrome (PCOS). Pak. J. Med. Sci. Q..

[70] Shaman A.A., Mukhtar H.B., Mirghani H.O. (2017). Risk factors associated with metabolic syndrome and cardiovascular disease among women with polycystic ovary syndrome in Tabuk, Saudi Arabia. Electron. Physician.

[71] Shishehgar F., Ramezani Tehrani F., Mirmiran P., Hajian S., Baghestani A.R. (2016). Comparison of the association of excess weight on health related quality of life of women with polycystic ovary syndrome: An age- and BMI-matched case control study. PLoS ONE.

[72] Sidra S., Tariq M.H., Farrukh M.J., Mohsin M. (2019). Evaluation of clinical manifestations, health risks, and quality of life among women with polycystic ovary syndrome. PLoS ONE.

[73] Smith J., Ayre J., Jansen J., Cvejic E., McCaffery K.J., Doust J., Copp T. (2021). Impact of diagnostic labels and causal explanations for weight gain on diet intentions, cognitions and emotions: An experimental online study. Appetite.

[74] Talpur D.N., Shaikh D.D., Dalwani D.N., Ghori D.A., Hanif D.S., Memon D.K. (2023). Frequency of polycystic ovarian syndrome (PCOs) in females presenting with infertility. J. Popul. Ther. Clin. Pharmacol..

[75] Varadan M., Gopalkrishna P., Bhat P.V., Kamath S.U., Thriveni G.K., Kumar S. (2019). Influence of polycystic ovary syndrome on the periodontal health of Indian women visiting a secondary health care centre. Clin. Oral Investig..

[76] Varanasi L.C., Subasinghe A., Jayasinghe Y.L., Callegari E.T., Garland S.M., Gorelik A., Wark J.D. (2018). Polycystic ovarian syndrome: Prevalence and impact on the wellbeing of Australian women aged 16–29 years. Aust. N. Z. J. Obstet. Gynaecol..

[77] Vutyavanich T., Khaniyao V., Wongtra-Ngan S., Sreshthaputra O., Sreshthaputra R., Piromlertamorn W. (2007). Clinical, endocrine and ultrasonographic features of polycystic ovary syndrome in Thai women. J. Obstet. Gynaecol. Res..

[78] Wang W., Zeng W., He S., Shi Y., Chen X., Tu L., Yang B., Xu J., Yin X. (2023). A new model for predicting the occurrence of polycystic ovary syndrome: Based on data of tongue and pulse. Digit. Health.

[79] Yan D., Yan-Fang W., Shi-Yang Z., Rui-Lin M., Xue-Song D., Xiao M., Wei X., Aijun S. (2021). Is polycystic ovary syndrome appropriately diagnosed by obstetricians and gynaecologists across China: A nationwide survey. J. Ovarian Res..

[80] Zhang H.Y., Guo C.X., Zhu F.F., Qu P.P., Lin W.J., Xiong J. (2013). Clinical characteristics, metabolic features, and phenotype of Chinese women with polycystic ovary syndrome: A large-scale case-control study. Arch. Gynecol. Obstet..

[81] Zhao Y., Qiao J. (2013). Ethnic differences in the phenotypic expression of polycystic ovary syndrome. Steroids.

[82] Alur-Gupta S., Dokras A., Cooney L.G. (2024). Management of polycystic ovary syndrome must include assessment and treatment of mental health symptoms. Fertil. Steril..

[83] Chaudhari A.P., Mazumdar K., Mehta P.D. (2018). Anxiety, depression, and quality of life in women with polycystic ovarian syndrome. Indian J. Psychol. Med..

[84] Li S.J., Zhou D.N., Li W., Yang J. (2017). Mental health status assessment in polycystic ovarian syndrome infertility patients: A pilot study. J. Huazhong Univ. Sci. Technol. Med. Sci..

[85] Chemerinski A., Cooney L., Shah D., Butts S., Gibson-Helm M., Dokras A. (2020). Knowledge of PCOS in physicians-in-training: Identifying gaps and educational opportunities. Gynecol. Endocrinol..

[86] Sacca L., Okwaraji G., Densley S., Marciniak A., Knecht M., Wilson C., Pilitsis J.G., Kimberly Hopkins D. (2024). Polycystic ovary syndrome and chronic pain among females and individuals of childbearing age: A scoping review. SAGE Open Med..

[87] Martin M.L., Halling K., Eek D., Krohe M., Paty J. (2017). Understanding polycystic ovary syndrome from the patient perspective: A concept elicitation patient interview study. Health Qual. Life Outcomes.

[88] Lu K.T., Ho Y.C., Chang C.L., Lan K.C., Wu C.C., Su Y.T. (2022). Evaluation of bodily pain associated with polycystic ovary syndrome: A review of health-related quality of life and potential risk factors. Biomedicines.

[89] Women with PCOS Should Be Screened for Mental Health Disorders. Endocrinology.org. https://www.endocrinology.org/press/press-releases/women-with-pcos-should-be-screened-for-mental-health-disorders/.

[90] Gibson-Helm M., Teede H., Dunaif A., Dokras A. (2016). Delayed diagnosis and a lack of information associated with dissatisfaction in women with polycystic ovary syndrome. J. Clin. Endocrinol. Metab..

[91] Teede H.J., Tay C.T., Laven J.J.E., Dokras A., Moran L.J., Piltonen T.T., Costello M.F., Boivin J., Redman L.M., Boyle J.A. (2023). Recommendations from the 2023 International Evidence-based guideline for the assessment and management of polycystic ovary syndrome. J. Clin. Endocrinol. Metab..

[92] Kalra S., Vaidya R., Verma M., Joshi A. (2023). Primary care screening tool for polycystic ovary syndrome: Step one in the battle against non-communicable disease. Indian J. Endocrinol. Metab..

[93] Boivin M.J., Fatehi F., Phillips-Chan A.E., Richardson J.R., Summers A.N., Foley S.A. (2020). Exploratory study of a screening measure for polycystic ovarian syndrome, quality of life assessment, and neuropsychological evaluation. BMC Womens Health.

[94] Lau G.M., Elghobashy M., Thanki M., Ibegbulam S., Latthe P., Gillett C.D., O’Reilly M.W., Arlt W., Lindenmeyer A., Kempegowda P. (2022). A systematic review of lived experiences of people with polycystic ovary syndrome highlights the need for holistic care and co-creation of educational resources. Front. Endocrinol..

[95] Islam H., Masud J., Islam Y.N., Haque F.K.M. (2022). An update on polycystic ovary syndrome: A review of the current state of knowledge in diagnosis, genetic etiology, and emerging treatment options. Womens Health.

[96] Moran L.J., Brown W.J., McNaughton S.A., Joham A.E., Teede H.J. (2017). Weight management practices associated with PCOS and their relationships with diet and physical activity. Hum. Reprod..

[97] Cowan S., Lim S., Alycia C., Pirotta S., Thomson R., Gibson-Helm M., Blackmore R., Naderpoor N., Bennett C., Ee C. (2023). Lifestyle management in polycystic ovary syndrome—Beyond diet and physical activity. BMC Endocr. Disord..

[98] Zehravi M., Maqbool M. (2016). Polycystic ovary syndrome and infertility: An update. Int. J. Adolesc. Med. Health.

