# Supplementary material for: Assessment of the Validity and Quality of Polycystic Ovarian Syndrome (PCOS) Screening Tools Available for Women Globally: A Systematic Review
Source: Clin Pract. 2024 Aug 23;14(5):1625–49. doi: 10.3390/clinpract14050131 (PMC11417729; doi:10.3390/clinpract14050131)
Supplement: Supplementary file 1 [file clinpract-14-00131-s001.zip › clinpract-3165212-supplementary.pdf]

Article No.	Primary Author/Year	Country	Study Design	Sample Size	Study Population	Age Range	Study Purpose	Constructs Measured	Type of SDOH	Status of PCOS Diagnosis at Start of Study
**1**	Böttcher et al., 2017	Germany	Case-control study	n=199 cases and n=199 controls	German-speaking patients diagnosed with PCOS and recruited by outpatient department of the University Clinic of Gynecologic Endocrinology and Reproductive Medicine Innsbruck, Austria, between 2012 and 2014.	18-45 years old	To validate the PCOSQ-G HRQoL in determining if patients with PCOS have higher rates of depression and anxiety with a reduced PCOSQ.	Medical history, demographic data, PCOSQ status (emotion, hirsutism, weight (BMI), infertility, and menstruation), mental health (anxiety and depression), and SF-36 status (physical function: physical role function, bodily pain, and general health; psychological function: vitality, social function, emotional role function, and mental health), semen analysis of partners.	Age, marital/partnership status, education.	Completed
**2**	Conway et al., 2014	Countries acting as members of ESE	Cross-sectional study	n=357	ESE members who answered the questionnaire on the ESE website between June 2012 and April 2013 and those who attended the 15th European Congress of Endocrinology held in Copenhagen, Denmark, April 27th– May 1st, 2013.	24-80 years old	To examine current practices for diagnosis and management of PCOS by specialists across Europe.	General characteristics of patients with PCOS seen by endocrinologists, most commonly used diagnostic criteria for PCOS, the biochemical parameters used in the differential diagnosis of hyperandrogenism, long-term concerns about patients, and most common treatment choices.	Age, gender, employment.	Not completed

**4**	Ding et al., 2022	China	Retrospective cohort study	n=800 (n=400 cases and n=400 controls)	Adolescents with PCOS who attend the gynecology clinic of the Affiliated Hospital of Zunyi Medical University from January 2022 to August 2022.	10-19 years old	To calculate the absolute risk of depression outcomes in adolescents with PCOS and develop a prediction model to identify at-risk populations.	Family history of psychiatric illness, duration of illness, personality (via JEPQ), emotional regulation skills, medication use, duration of medicine use, sleep (via AIS), interpersonal sensitivity, social support, clinical symptoms (hirsutism, acne, body image), illness perception, lab findings (SHBG, FAI, E2, FSH, LH, progesterone, prolactin, total testosterone, dehydroepiandosterone sulphate, third-generation thyrotropin, insulin), CDI status (anhedonia, poor efficacy, low self-esteem, negative emotions, interpersonal problems), BMI, WHR.	Age, nationality, education, living conditions, single-parent family.	Completed
**5**	Dou et al., 2016	China	Protocol study	n=410	Female patients of childbearing age diagnosed with PCOS at the Reproduction and Genetic Center of Peking University First Hospital from June 2015 to January 2016.	16-40 years old	To explore the accuracy and best cut-off points of three obesity parameters, BMI, WC, and PBF, in PCOS diagnosis and compare sensitivity and specificity in order to use them in the prediction, screening, and diagnosis of PCOS in high-risk population.	Anthropometric measurements (height, weight, BMI, WC, body composition, and PBF).	Age.	Completed

**6**	Duman et al., 2021	Turkey	Case-control study	n=65 (n=30 cases and n=35 controls)	Female patients admitted to the endocrinology outpatient clinic with PCOS-like complaints and subsequently diagnosed with PCOS via Rotterdam criteria.	17-44 years old	To examine the relationship between Drosha, XPO5, and Dicer, which are involved in miRNA formation, with PCOS.	Demographic data, BMI, pelvic ultrasound, menstrual patterns/irregularities, lab measurements (FSH, LH, E2, free T4, prolactin, 11-deoxycortisol, androstenedione, total testosterone, DHEA-SO4, 17-OHP, TSH, vitamin D, insulin, and glucose levels).	Age.	Not completed
**7**	Guyatt et al., 2004	Canada, USA, UK	Randomized control trial study	n=393	Female patients with PCOS at tertiary care sites.	29.4 +-5.7 years old	To explore the measurement properties of the PCOSQ and assess the effectiveness of troglitazone for the treatment of the PCOS.	PCOSQ status (emotion, hirsutism, weight, infertility, and menstruation), measures of body and facial hair growth (Ferriman–Gallwey and microscopic examination), menstrual cyclicity, free testosterone levels.<br><br>Emotions domain: questions about feeling depressed, easily tired, worried, moody, having low self-esteem, being self-conscious, and fearful of getting cancer.	N/A.	Completed
**8**	Hariprasath et al., 2023	India	Mixed-methods study	n=365	South Indian females with PCOS.	18-40 years old	To assess the impact of various clinical features of PCOS on the HRQoL of South Indian women including an additional social issues domain of HRQoL.	PCOSQ status (emotion, hirsutism, weight, infertility, and menstruation) and social issues faced (social anxieties/pressures).	Age, education, marital status, occupation, SES.	Completed

**9**	Hollinrake et al., 2007	USA	Cohort study	n=206 (n=103 cases and n=103 controls)	Women with PCOS seen at the University of Iowa Hospitals and Clinics between May 2004 and August 2005; women without PCOS seen during the same time period for an annual exam were used as control subjects.	18-50 years old	To estimate the prevalence of depressive disorders in women with PCOS and to determine the role of androgens and other metabolic markers associated with PCOS in the development of depression.	PRIME-MD PHQ status (mental health), BDI status (depression), demographics, medical history (medications, obstetric history, personal history of psychiatric illness and counseling or treatments, family history of psychiatric illness), BMI, if currently attempting to get pregnant.	Age, race, education, employment status, marital status, cigarette, marijuana, and alcohol use.	Completed
**10**	Hussain et al., 2015	India	Cross-sectional study	n=150 (n=110 cases and n=40 controls)	Women with PCOS in the Kashmir Valley at the outpatient department at the Sher-e-Kashmir Institute of Medical Sciences.	16-40 years old	To investigate the prevalence of psychiatric disorders among women in ambulatory treatment for PCOS.	Menstrual history (age of menarche, regularity, duration, number of cycles per year), PMH (past or current history of psychiatric disorder or morbidity).	Age.	Not completed
**11**	Jedel et al., 2008	Sweden	Randomized control trial study	n=84	Women with PCOS enrolled in the study November 2005 through September 2008 at a baseline of moody, low self-esteem, being self-conscious, and fear of getting cancer as a result of having PCOS.	18-37 years old	To determine the test–retest reliability and confirm the domain structure of the Swedish version of PCOSQ.	PCOSQ status (emotion, hirsutism, weight, infertility, and menstruation), hirsutism (via Ferriman–Gallwey), hormones (testosterone, DHEA-SO4, SHBG), anthropometrics (weight, height, BMI, WHR).	Age.	Completed
**12**	Jedel et al., 2010	Sweden	Case-control study	n=60 (n=30 cases and n=30 controls)	Women with PCOS recruited by the Sahlgrenska Academy, University of Gothenburg, Sweden, between November 2005 and September 2008.	16-40 years old	To compare symptoms of anxiety and depression in women with PCOS and controls matched for age, body weight, and BMI.	Gynecological examination and vaginal ultrasound to confirm PCOS status, anthropometric data (weight, height, BMI, WHR), menstrual history, hormones (testosterone and SHBG), CPRS-S-A status (psychiatric symptoms).	Age, marital status, biological children, employment status and sick leave, exercise frequency, cigarette smoking, alcohol use.	Completed

**13**	Jones et al., 2004	UK	Cross-sectional study	n=92	Women of reproductive age with PCOS recruited from an outpatient gynecology clinic at the Jessop Wing, Royal Hallamshire Hospital, Sheffield.	20-41 years old	To evaluate the other psychometric properties of the PCOSQ, in particular the reliability, validity, and factor structure of the domains when assessing the HRQoL in women with PCOS.	PCOSQ status (emotion, hirsutism, weight, infertility, and menstruation), PCOS clinical features (acne, hormones (LH/FSH ratio, testosterone, SHBG)), vaginal ultrasound to diagnose PCOS, BMI/	Ethnicity, age.	Completed
**14**	Joshi et al., 2021	India	Cross-sectional study	n=105	Women diagnosed with PCOS recruited by an outpatient department of gynecology of a general municipal hospital in India conducted over a period of one year.	18-50 years old	To study the prevalence of depression, anxiety, and body image disturbances and self-esteem in patients with PCOS and to study the relationship of depressive symptoms with self-esteem and body image disturbances.	BDI status (depression), HAM-D status (depression), HARS (anxiety), BICI status (body image perception), RSES status (self-esteem)/	Age, marital status, religion, occupational status, educational level.	Completed
15	Karjula et al., 2017	Finland	Cohort study	N= 2967 (controls and women with PCOS)	Northern Finland Birth Cohort 1966.	31 and 46 years old	To investigate the prevalence of anxiety/depression and their coexistence in women with PCOS/PCOS-related symptoms at ages 31 and 46.	PCOS status, anxiety, depression, BMI, and serum testosterone/FAI/	Age, SES, education.	Not completed
16	Karjula et al., 2020	Finland	Cohort study	N=1283 (controls and women with PCOS)	Northern Finland Birth Cohort 1966.	31 and 46 years old	To study generic HRQoL using the 15D questionnaire, life satisfaction, and self-reported health status in women with PCOS symptoms at ages 31 and 46 years.	HRQoL, PCOS status, anxiety, depression, BMI, serum testosterone/FAI, infertility, smoking, alcohol consumption, marital status/	Age, SES, education.	Not completed
17	Karjula et al., 2021	Finland	Cohort study	N=2269 (controls and women with PCOS)	Northern Finland Birth Cohort 1966.	31-50 years old	To investigate whether women with PCOS are at higher risk for psychotic disorders.	Psychosis, parental history of psychosis, BMI, hyperandrogenism, serum testosterone/FAI, SES.	Age, SES, education.	Not completed

18	Klipstein et al., 2006	United States	Cross-sectional study	N=78	PCOSA conference attendees.	23-40 years old	To investigate the hypothesis that an intrinsic association may exist between PCOS and bipolar disorder, independent of pharmacotherapy.	Bipolar disorder, PMH, family history, BMI.	Race, age.	Completed
19	Kocak et al., 2022	Turkey	Cross-sectional study	N=131	Female patients in an outpatient obstetrics and gynecology clinic.	18-39 years old	To determine depression symptoms and QoL in women with PCOS.	PMH, family history, psychosocial, emotional, fertility, sexual function, obesity, menstrual disorders, hirsutism, coping.	Age, educational status, employment, marital status, income level perception, living place.	Completed
20	Kolahi et al., 2015	Iran	Cross-sectional study	N=200	Patients with PCOS in Isfahan Province.	15-41 years old	To investigate the relationship between QoL and coping strategies in patients with PCOS.	Coping strategies, acne, hirsutism, QoL, social protection, problem solving, cognitive and emotional.	Age, marital status, employment.	Completed
21	Kumarapeli et al., 2010	Sri Lanka	Case-control study	N=334 (controls and women with PCOS)	Women residing permanently in the district of Gampaha.	15-39 years old	To assess psychological distress, HRQoL, and their correlates.	PCOS status, PMH, hirsutism, acne, BMI, infertility.	Age, marital status, education, employment, SES.	Not completed
22	Lam et al., 2005	China	Cohort study	N=90	Hong Kong Chinese women who were referred to the Gynae-endocrinology Clinic in the PWH.	17-40 years old	To identify the characteristics of Hong Kong Chinese women with PCOS and to compare different diagnostic criteria.	Anovulation, hyperandrogenism, polycystic ovarian features on ultrasonography, LH predominance, obesity, IR, serum concentrations of FSH and E2 in the early follicular phase of an ovarian cycle, serum prolactin level, thyroid function test, serum concentrations of total testosterone, dyslipidemia.	Age.	Not completed

23	Lee et al., 2022	United States	Qualitative study	N=18 (physicians)	Gynecologists and PCPs at an urban academic medical center.	N/A	To identify barriers and facilitators to the implementation of evidence-based guidelines among gynecologists and PCPs caring for women with PCOS.	PCOS diagnosis, communication with patients, impact of PCOS diagnosis, management of PCOS.	Sex.	Not completed
24	Lerchbaum et al., 2013	Austria	Cross-sectional study	N=671	Women with PCOS at an endocrinology outpatient clinic.	16-45 years old	To investigate whether HbA1c and FG are useful in predicting the presence of prediabetes and T2DM in a large cohort of women with PCOS.	HbA1c, fasting glucose, BMI, blood pressure, serum lipids, total testosterone, SHBG, 2-hour glucose, medical and family history.	Age.	Completed
25	Lin et al., 2016	Taiwan	Cohort study	N=102	Women with PCOS in a medical center in southern Taiwan.	18-45 years old	To evaluate the responsiveness, longitudinal validity, and measurement invariance of the Chi-PCOSQ.	Two-hour postload glucose and insulin levels, BMI, WHR, blood pressure, HRQoL.	Age, gender, residence, education, and occupation.	Completed
26	Maleki et al., 2022	Iran	Cross-sectional study	N=100	Women with PCOS in Hamadan health centers.	18-45 years old	To assess predictive factors of sexual QoL among Iranian women with PCOS.	Psychosexual feeling, sexual relationship satisfaction, self-worthlessness, sexual repression, sexual compatibility, somatic symptoms, sleep and anxiety, depression, social incompatibility, hirsutism, abortion history, history of infertility, and BMI.	Age, education, and family income.	Completed

27	Mei et al., 2022	Malaysia	Cohort study	N=138	Women who were diagnosed with PCOS using Rotterdam criteria in a gynecology clinic.	18-45 years old	To develop a Mal-PCOSQ and to evaluate the health-related impact of Malaysian women with PCOS.	Emotion, body hair, weight, infertility, menstrual problems, BMI, physical functioning, role limitations due to physical problems, bodily pain, general health perception, social functioning, role limitations due to emotional problems, vitality, and mental health.	Age and ethnicity.	Completed
28	Mojahed et al., 2023	Iran	Case-control study	N=212	Married women with PCOS and their married family members.	Not available—mean age is 27 years old	To assess depression, sexual dysfunction, and sexual QoL in women with PCOS compared with healthy subjects.	Depression, sexual dysfunction, desire, lubrication, arousal, orgasm, satisfaction, and pain.	Age	Completed
29	Nasiri-Amiri et al., 2016	Iran	Cross-sectional study	N=200	Women with PCOS referred to the Reproductive Endocrinology Research Center.	18-40 years old	To develop a comprehensive instrument to assess the HRQoL of Iranian women with PCOS and to assess its psychometric properties.	PMH, BMI, psychosocial and emotional, fertility, sexual function, obesity and menstrual disorder, hirsutism, and coping.	Age, education, income, marital status.	Completed
30	Nasiri-Amiri et al., 2018	Iran	Cross-validation study	n = 350	Females with PCOS referred to the gynecology, endocrinology, and dermatology clinic of Babol University of Medical Sciences in Babol, Iran.	18-40 years old	To analyze exploratory and confirmatory factor structures of the HRQoL instrument in females with PCOS using the PCOSQ-50.	Demographic characteristics (height, weight, waist circumference, and hip circumference), reproductive and gynecological history, menstrual status, hyperandrogenic symptoms (acne, hirsutism, and alopecia), physical appearance, infertility, chief complaint, medications, and family history.	Age, length of education, income, marital status, SES.	Completed

31	Neubronner et al., 2021	Singapore	Cross-sectional cohort study	n = 389	Healthy women recruited at an annual health screening for hospital staff and volunteers from a university community, and PCOS cases referred to tertiary gynecology clinics in Singapore.	21-45 years old	To identify if excess body weight affects menstrual cycle length, excessive hair growth, and other phenotypic features in healthy women without PCOS and then to assess whether having PCOS exacerbates the effects of high BMI.	Demographic characteristics (height and weight for BMI), reproductive health history (menstrual cycle profiling and obstetric history), anthropometric evaluation, ovary evaluation via transvaginal ultrasound, blood sampling for reproductive hormones and metabolic markers on days 2 and 5 of the menstrual cycle, and hair growth.	Age, race, marital status, employment status, income, and smoking status.	Not completed
32	Ning et al., 2013	USA (IRB is in Johns Hopkins)	Retrospective cross-sectional study	n = 262	IVF-focused website linking doctors and specialists in IVF centers around the world.	N/A	To determine what diagnostic values/tools practitioners are utilizing to diagnose PCOS.	Demographic features of the clinic and IVF unit.<br><br>Features used by practitioners to diagnose PCOS including Rotterdam criteria, measuring LH/FSH ratio, AMH, and androgens; ultrasound appearance of polycystic ovaries in presence of anovulation with normal prolactin; nonclassical congenital adrenal hyperplasia; and other features related to diagnosing PCOS.	N/A.	N/A

33	Ou et al., 2015	Taiwan	Cross-sectional study	n = 80	Females from the National Cheng Kung University Hospital diagnosed with PCOS who were regularly followed at an outpatient clinic.	18-45 years old	To develop the Chi-PCOSQ.	Demographic characteristics (age, gender, residence, highest education level, family history of diabetes, disease duration, and disease subtype).<br><br>Height, weight, BMI, WC and hip circumference, acne, hair loss, and blood pressure.<br><br>PCOSQ—QoL impairment regarding PCOS and emotion, hair growth, body weight, infertility, and menstruation.<br><br>WHOQOL-BREF—assessed physical health, psychological health, social relations, and environment.<br><br>EQ-5D—measured mobility, self-care, usual activities, pain/discomfort, and anxiety/depression.	Age, educational level, and smoking status.	Completed

34	Panico et al., 2017	Italy	Cross-sectional study	Women with PCOS: n = 100<br><br>Healthy healthy-weight control group: n = 40<br><br>Healthy obese control group: n = 40	Females were recruited and placed into 3 categories: those with a prior diagnosis of PCOS, those with a healthy and normal weight as one control group, and those who were healthy and obese as another control group.	Women with PCOS: 17.2-29 years old<br><br>Healthy healthy-weight control group: 16.4-31.8 years old<br><br>Healthy obese women control group: 17.1-29.5 years old	To evaluate if PCOS alone affects the patients' QoL and to what extent obesity contributes to worsening this disease.	The domains of 3 surveys were measured:<br><br>SCL-90-R: somatization, obsessive–compulsive, interpersonal, sensitivity, depression, anxiety, aggression, phobia, paranoid ideation, psychoticism, and sleep disorders.<br><br>SF-36: physical function, physical role function, bodily pain, general health, vitality, social function, emotional role function, and mental health.<br><br>PCOSQ: emotions, hirsutism, body weight, infertility, and menstrual disorders.	Age.	Completed
35	Patil et al., 2022	India	Descriptive observational study	n = 226	Females were recruited based on awareness of interventional programs related to PCOS. Individuals with symptoms similar to those described for PCOS were screened, and the ones diagnosed with PCOS ultimately took part in the study.	Mean age: 26.27 ± 4.9 years	To comprehensively address the wide spectrum of PCOS by creating a multidisciplinary model of care to screen and manage multifaceted manifestations of PCOS, and to diagnose and treat associated comorbidities such as metabolic syndrome, dermatologic manifestations, and psychological issues.	Detailed history including height, weight, BMI, waist–hip ratio, FG score, acne, and acanthosis nigricans.<br><br>Ultrasound of the pelvis and abdomen to detect fatty liver and cholelithiasis.<br><br>Hormonal tests to look at TSH, prolactin, 17-OHP, LH, FSH, testosterone, and SHBG.<br><br>Screenings for emotional, mental health, and QoL .	Age, marital status, education status, working status.	Not completed

36	Patten et al., 2023	Australia	Secondary analysis of a two-arm randomized clinical trial	n = 29	Females with PCOS were recruited via community and social media advertisements.	18-45 years old	To determine the efficacy of HIIT in comparison to MICT for improving mental health and QoL in women with PCOS.	Depression, anxiety, stress, HRQoL, anthropometric measurements, aerobic capacity, and insulin sensitivity.	Age.	Completed
37	Petkova et al., 2018	Bulgaria	Pilot study	n = 24	Females with PCOS recruited from pharmacies in Sofia, Bulgaria, based on referrals from pharmacists and their disease dossier.	15-35 years old	To assess the impact of PCOS on the QoL of Bulgarian patients via the PCOSQ.	Sociodemographic information including age, disease duration, and BMI.<br><br>PCOSQ—measured 5 domain topics including body hair, emotions, overweight, menstrual problems, and infertility.	Age.	Completed
38	Prathap et al., 2018	India	Cross-sectional study	n = 64	Females with PCOS attending the OB/GYN outpatient department at a medical college and hospital in Kerala, India.	15-35 years old	To assess the proportion of anxiety and depression and the QoL and to find the correlates of QoL in women with PCOS.	Sociodemographic data, family history of psychiatric illness, any psychiatric illness/treatment in husband, age and gender of children, hirsutism, depression, anxiety, and QoL.	Age, religion, educational status, occupational status, marital status, and income.	Completed
39	Radwan et al., 2023	Saudi Arabia	Cross-sectional study	n = 155	Females with PCOS living in Jeddah, Saudi Arabia, recruited from the King Abdulaziz University Hospital.	18-47 years old	To investigate the association between PCOS and junk food consumption in women of childbearing age.	Sociodemographic information, medical history, PCOS symptoms, and junk food consumption patterns.	Age, marital status, family income, parents' educational level, partner's educational level, and smoking status.	Completed
40	Rasgon et al., 2005	USA, Germany, Netherlands, and Chile	Cross-sectional study	n = 80	Females who were being treated in an outpatient setting for BP-I, BP-II, or BP-NOS and who were receiving long-term treatment with an antimanic agent were recruited from 4 national and 2 international websites within	18-45 years old	To assess whether women treated with divalproex sodium or another treatment for bipolar disorder would exhibit differential rates of menstrual abnormalities, differential rates of hormonal or metabolic abnormalities, and differential rates of	Hormone values, information on past medical, psychiatric, and reproductive health history including menstrual history, family history of menstrual/reproductive problems, and family psychiatric history.<br><br>Other constructs included assessment of hirsutism, height, weight, and acne.	Age.	Not completed

					the Stanley Foundation Treatment Network.		PCOS in association with treatment.			
41	Robinson et al., 2020	USA	Longitudinal cohort study	n = 1915	Mothers with self-reported PCOS and hirsutism and their children from the Upstate KIDS cohort took part in the study. This cohort consisted of infants conceived using fertility treatments and multiple births.	Mean age: 31.3 ± 5.9 years	To study the associations between maternal PCOS and hirsutism with offspring ADHD, anxiety, conduct disorder, and behavioral issues.	Baseline questionnaire assessed: reproductive history, familial health status, and sociodemographic information such as parental age, height, weight, history of affective disorders, maternal race/ethnicity, education, insurance type, marital status, parity, history of gynecological medical conditions, history and type of infertility treatment, and smoking use during pregnancy.<br><br>Information from birth certificates on the child’s sex and plurality, and maternal prepregnancy height and weight.<br><br>Follow-up annual questionnaire: questions to the mothers about their children’s development, as well as their children and any ADHD, anxiety, conduct disorder, or medication use.	Maternal age, ethnicity/race, education, insurance, and marital status.<br><br>Paternal and child ages.	Not completed

								Mothers rated their child's behaviors by assessing emotional symptoms, peer relationship problems, conduct problems, hyperactivity/inattention, and prosocial behaviors.		
42	Rodrigues et al., 2012	Brazil	Cross-sectional study	n = 166	Females with PCOS, and for each woman with PCOS, another control without PCOS was taken, matched for the same age, educational status, and presence/absence of sexual partners.	18-30 years old	To evaluate the prevalence of common mental disorders in women diagnosed with PCOS as compared with paired controls without this syndrome.	Height and weight, a questionnaire to measure sociodemographic data, and self-reporting questionnaire to measure indications of common mental disorders (anxiety, mood, and somatoform disorders).	Age, educational level of household head, and race.	Not completed
43	Rodriguez et al., 2020	United States	Pilot study	n= 9	Virtual test subjects were generated by creating answers to preformulated questions built	≥16 years old	To examine the function of the irregular cycle feature on the Clue app, as well as to determine the sensitivity and	Height, weight, menstrual cycle length, duration of flow, menstruation-related pain symptoms, and method of birth control.	Age.	Not completed

					into the irregular cycle feature.		specificity of the feature prior to testing with human subjects.			
44	Rzo'nca et al., 2018	Poland	Cross-sectional study	n= 504 (n=250 with PCOS and n=254 control)	Female patients using healthcare services (primary care, specialist outpatient care, and inpatient/hospital care) in Lublin, Podkarpacie, Pomerania, and Greater Poland Provinces.	≥18 years old	To identify differences in QoL and SwL for women with PCOS compared to healthy controls. To identify factors involved in determining QoL and SwL in women with PCOS.	PCOS status, QoL, SwL, BMI, having children, time from PCOS diagnosis, and professional activity.	Age and socioeconomic standing.	Complete
45	Salva-Pastor et al., 2020	Mexico	Cross-sectional study	n=98	Female patients from the gynecology services of Manuel Gea Gonzalez and Medica Sur hospitals in Mexico City.	18-44 years old	To identify the frequency of NAFLD in Mexican women with PCOS compared to healthy controls.	BMI, PCOS, CAP, LSM.	Age.	Complete
46	Sánchez-Ferrer et al., 2017	Spain	Case-control Study	n= 285 (n=126 with PCOS and n=159 control)	Female patients from the gynecology services of Virgen de la Arrixaca University Clinical Hospital in Murica, Spain.	18-40 years old	To examine the correlation between AGD measurements, serving as a biomarker of prenatal androgen exposure and PCOS in Mediterranean women.	AGD_{AC}, AGD_{AF}.	Age and ethnicity.	Complete
47	Santos et al., 2022	Brazil	Randomized controlled clinical trial	n=23 (n=12 in HIIT group and n=11 in control group)	Female patients at the Gynecology and Endocrinology Clinic of the Januario Cicco Maternity School during medical consultations, as well as on social networks and	18-40 years old	To examine how HIIT and detraining affect the QoL and mental health of women with PCOS.	Weight, height, age at onset of menarche, and the number of menstrual cycles per year, presence of acne and/or hirsutism, and medical illness, use of any medication, self-esteem, depression, satisfaction with body parts.	Age, place of residence, parents' profession and educational status, monthly income, family structure.	Not completed

					through the SIGAA.				
48	Sari et al., 2020	Turkey	Case-control study	n=87 (n=50 with PCOS and n=37 control)	Female patients in The Department of Child and Adolescent Psychiatry and the Department of Pediatric Endocrinology outpatient clinic of Cumhuriyet University in Sivas, Turkey.	12-18 years old	To examine body perception, self-esteem, and comorbid psychiatric diseases in women with PCOS compared to healthy controls.	BMI and IR.	Age, family structure, education, place of residence, parents' profession and educational status, and monthly income.	Not completed
49	Sayyah-Melli et al., 2015	Iran	Case-control study	n= 1,540 (n=742 with PCOS and n=798 control)	Female patients at the clinics of Tabriz University of Medical Sciences.	Mean cases group 23.5 (SD of 5.2); mean control group age is 27.1 (SD of 5.9)	To examine the correlation between psychopathology and social variables between PCOS patients and controls.	BMI.	Age, marital status, education, and, SES, employment.	Complete
50	Scaruffi et al., 2014	Italy	Case-control study	n=94 (n=49 with PCOS and n=45 control)	Female patients from the Endocrine Unit of St. Orsola–Malpighi Hospital–University of Bologna and the Clinical Medical 3 of Health Centre/Hospital–University of Padua.	19-41 years old	To examine if PCOS, a hyperandrogenic syndrome, characterizes a strong psycho(patho)logical personality.	Weight, BMI, testosterone levels, LH/FSH ratio, IR, and glucose tolerance, glucose, insulin, LH, FSH, E2, testosterone, DHEA-SO4, 17-OHP, and androgen levels in the blood plasma, reduced coping abilities and social skills, depression, perceptual distortion and cognitive slippage, constant alertness and worry, at risk of suicide, chronic stress.	Age and education.	Not completed

51	Scaruffi et al., 2018	Italy	Case-control study	n=97 (n=59 with PCOS and n=38 control)	Outpatient females at the two gynecological endocrinology services of the University Hospital Citta della Scienza e della Salute.		To examine personality characteristics, body image, and alexithymia in women with PCOS.	BMI, difficulty in identifying feelings, alexithymia, body perception, hypochondriasis, depression, hysteria, psychopathic deviation, paranoia, psychasthenia, schizophrenia, and social introversion.	Age and education.	Complete
52	Shakil et al., 2020	Pakistan	Correlational study	n=60	Female patients from government hospitals in Lahore, Pakistan.	18-38 years old	To examine if sexual dysfunction contributes to the development of depressive symptoms and life dissatisfaction in women with PCOS.	Sexual functioning, life satisfaction.	Age, education, family structure, employment status, income/SES.	Complete
53	Shaman et al., 2017	Saudi Arabia	Cross-sectional study	n=404	Female infertile patients at an infertility clinic of King Khalid Hospital.	15-45 years old	To identify global CVR and prevalence of MS in women with PCOS.	Weight, WC, BMI, HDL, LDL, TC, TG, FBS, systolic BP, diastolic BP.	Age and ethnicity.	Not completed
54	Shishehgar et al., 2016	Iran	Case-control study	n=282 (n=142 with PCOS and n=140 control)	Female patients receiving their annual gynecological exam at gynecologic centers affiliated with Shahid Beheshti University of Medical Sciences in Tehran.	18-40 years old	To identify the relationship between BMI and domain scores of SF-36 in women with PCOS, and to compare these correlations between PCOS and control groups.	HRQoL, BMI, menstrual regularity, WC, Ferriman–Gallwey scores of hirsutism.	Age, education, marital status parity.	Complete

55	Sidra et al., 2019	Pakistan	Cross-sectional study, observational study	n= 440 patients	Female patients of reproductive age admitted to numerous Pakistani hospitals.	15-44 years old	To identify the impact of PCOS complications on QoL and prescribing practices in healthcare centers across Pakistan.	Demographic and clinical characteristics: weight, age, major disease (PCOS and PCOS w/ other endocrine disorders), FBG level to test for DM, ultrasound test for polycystic ovaries, menstrual flow, hirsutism, acne, depression, infertility, comorbidities such as HTN, cardiac disease, and other endocrine disorders, QoL assessment.<br>QOL in PCOS patients compared to clinical aspects measured: DM, obesity, HTN, hyperandrogenism, IR, risk of endometrial cancer. and sleep apnea.<br>Treatments for PCOS patients: drug–drug interactions, prescribed metformin, infertility drugs.<br>Measurements of ADR and prescribed drug use: vomiting, diarrhea, uterine bleeding.	Age, gender, marital status.	Completed
56	Smith et al., 2021	Australia	Experimental study	n= 545 females	Females of reproductive age recruited through Dynata.	18-45 years old	To experimentally test the influence a PCOS disease label vs. overweight or no disease has on the intention of healthy eating and personal weight control and investigate the possible impact of genetic and environmental factors.	Baseline measures: self-perceived weight and 4 items related to healthy eating perception.<br>Primary outcomes: 3 items related to intention to eat healthier, 3 items to perceived personal control of weight.<br>Secondary outcomes: 3 items related to weight stigma; single item for blameworthiness, worry, and perceived anxiety;	Gender, age, location, interests.	Not completed

								8 items for illness perceptions; Rosenberg self-esteem scale to assess self-esteem; 8 items for belief diet will reduce health risks; and 1 item for menu item choice.		
57	Talpur et al., 2023	Pakistan	Descriptive study, cross-sectional study	n= 88 patients	Female patients with primary infertility from a Pakistani medical center.	18-35 years old	To identify the frequency of PCOS in females with primary infertility.	Obesity, medical history (HTN, DM), primary infertility.	Age, duration of marriage, gender.	Completed
58	Varadan et al., 2019	India	Case-control study	n = 60 patients	30 female patients with PCOS and 30 healthy patients reporting to Indian Department of OB/GYN.	18-45 years old	To identify the possible association of PCOS on periodontal health.	Demographic features such as weight, height, and BMI. Features used by physicians to diagnose PCOS including Rotterdam criteria, biochemical parameters (TG, FBS, FI). Periodontal parameters included plaque index by Silness and Loe, mGI by Lobene, probing depth and bleeding on probing MDA analyzed using Kei Satoh's method, and MPO analyzed using method by Matheson et al.	Age, duration of marriage, gender.	Completed
59	Varanasi et al., 2018	Australia	Cross-sectional study	n= 300 females	Females of reproductive age recruited through social media advertisements and YFHI and Safe-D online questionnaires.	16-29 years old	To identify the prevalence of PCOS in women using NIH criteria compared to self-reported diagnosis in order to compare comorbidities between the two.	PCOSQ—QoL impairment regarding PCOS and emotion, body weight, infertility. Demographic features: weight, height, BMI, WC and hip circumferences, sitting BP, acne, hirsutism, hormonal contraception, HTN, TC, history of pregnancies. Laboratory measurements: Fasting blood samples to test DHEA-SO4, SHBG, and total testosterone. Screening for anxiety	Age, gender.	Completed

								and depressive symptoms using K10.		
60	Vutyavanich et al., 2007	Thailand	Cross-sectional study	n = 62 patients	Female patients who attended outpatient gynecologic and infertility clinics.	18-40 years old	To identify the prevalence of PCOS in Thai female patients and to examine their hormonal features and ovarian ultrasound appearances.	Demographic characteristics: weight, BMI, leukorrhea, medical, surgical, and menstrual history, hyperandrogenism. Transvaginal ultrasound performed if presented with oligomenorrhea plus symptoms of acne, oily face, hirsutism. Use of transabdominal approach to women with no prior sexual exposure. Hormonal blood tests to measure prolactin, total testosterone, FSH and LH, DHEA-SO4, and 17-OHP ACTH stimulation test. Use of NIH criteria for PCOS diagnosis.	Age, gender, ethnicity.	Completed

61	Wang et al., 2023	China	Case-control study, retrospective study	n= 486 patients	A total of 285 PCOS patients from a Chinese hospital and 201 healthy female volunteers from the hospital's Physical Examination Center.	20-44 years old (the study corrected baseline data to 28)	To establish a machine learning model based on lingual pulse characteristic parameters as a noninvasive method to predict the onset of PCOS.	Demographic features: height, weight, BMI. Medical history: oligo- and/or anovulation, clinical and/or biochemical signs of hyperandrogenism and polycystic ovaries. Rotterdam criteria used for PCOS diagnosis. Tongue and pulse diagnosis data using TDA-1 tongue diagnosis instrument and PDA-1 pulse diagnosis instrument by Shanghai University of TCM research team. Tongue body and coating values of PCOS vs. healthy women. MLP classifier, XGBoost classifier, and SVM algorithms to measure and compare tongue and pulse parameters for AI-driven predictions.	Age.	Completed
62	Yan et al., 2021	China	Cross-sectional study	n= 2,328 OB/GYN MDs	A total of 2,200 practicing general OB/GYNs and 128 practicing re-OB/GYNs across China.	Approx. 24-56 years old	To describe the diagnostic criteria used and their application accuracy in the practice of PCOS caring among Chinese OB/GYNs.	Demographic characteristics: hospital classification, years in post, years involved in PCOS treatment, and the number of patients with PCOS treated annually. Use of AE-PCOS criteria demonstrating symptoms of oligo-menstruation, clinical hyperandrogenism, and biochemical hyperandrogenism. Rotterdam criteria, NIH criteria, and other criteria unknown. General OB/GYNs and Re-OB/GYNs and their frequency in the use of criteria items (never,	Age, gender, occupation.	Completed

								rarely, sometimes, often).		
63	Zhang et al., 2012	China	Case-control study	n=1,404 patients	A total of 719 women diagnosed with PCOS and 685 healthy women were sampled from a large-scale clinic in China.	18-45 years old	To investigate the clinical and biochemical features of a large-scale clinic based on the samples of Chinese women and to evaluate the value of Rotterdam criteria on PCOS.	Demographic features: BMI, obesity, height, weight, hirsutism, acne, HOMO-IR, menstrual cycle history, personal medical history, ovarian follicle number and volume, hormonal and metabolic parameters. Biochemical hyperandrogenemia, oligomenorrhea. Transvaginal ultrasound to detect polycystic ovaries. Fasting blood tests to measure FSH/LH, testosterone, E2, FI, FBS, 17-OHP, TSH, and ACTH.	Age, gender.	Completed

Table S1. Study characteristics.

Article No.	Primary Author/Year	Type of Methodology	Validated	Limitations of Measurement Tool Used	Mode of Administration	Analysis Used	Measured Associations

1	Böttcher et al., 2017	PCOSQ, HADS, German version of SF-36.	Yes	• Low response rate (30%).<br>•The HRQOL does not capture all of the associated comorbidities of PCOS.<br>• Proportion of patients with infertility problems or specific impairments may be larger than general population.<br>• Four weeks for the retest reliability is long and therefore prone to changes in individual health.<br>• Small sample size.	Personal interviews and self-administered questionnaires.	Chi-squared tests, corrected item–total correlation, receiver operating characteristic (ROC) curve, Pearson's correlation coefficients, t-tests, and effect size calculations (Cohen's d).	PCOSQ-G and HRQOL in German women.
2	Conway et al., 2014	Survey.	No	• Small sample size mainly composed of endocrinologists.<br>•Endocrinologists and OBGYNs utilize different definitions, diagnostic criteria, and treatment for PCOS.<br>•Endocrinologists across Europe diagnose PCOS differently.	Online survey.	Descriptive statistics and Student's t-test or Fisher's exact test.	PCOS diagnostic criteria and physician gender.
4	Ding et al., 2022	Kiddie-Schedule for Affective Disorders and Schizophrenia-Epidemiological version (K-SADS-E), Children's Depression Scale (CDI).	Yes	•Recall bias (online survey).<br>•Single-center study (limited sample).<br>•Inconsistency in diagnostic criteria among different ethnic groups (less generalizable).<br>•Patients treated with OCPs (can increase risk of depression) and metformin (can reduce depression), resulting in confounding results.<br>•Model was externally validated via temporal validation.	Personal interview (K-SADS-E), online questionnaire (CDI).	Student's t-test, Mann–Whitney U test, chi-squared test, Fisher's exact test, Pearson correlation coefficients, Spearman correlation coefficients, C statistic.	N/A.

5	Dou et al., 2016	Bioelectrical impedance body composition analyzer (multifrequency bioelectrical impedance analyzer NQA–PI); WHO anthropometric requirements.	No	N/A.	Measurements at outpatient clinic.	Independent sample t-test, Wilcoxon test, receiver operating characteristic curve (ROC curve) via Mann–Whitney method, AUC.	Obesity parameters in the diagnosis of PCOS in Chinese childbearing women.
6	Duman et al., 2021	Laboratory measurements.	No	N/A.	Labs drawn at hospital.	Kolmogorov–Smirnov test, Student's t-test, Mann–Whitney U test, Spearman's correlation test.	Enzyme levels (Drosha, Exportin-5 (XPO5), and Dicer) and PCOS diagnosis and phenotype.
7	Guyatt et al., 2004	PCOSQ, laboratory measurements.	Yes	•Limited generalizability due to lack of sociodemographic data.	Self-administered questionnaire.	Cronbach's α, Pearson correlation coefficients, factor analysis, principle components analysis, both unrotated and rotated (varimax rotation) analyses, analysis of covariance (ANCOVA).	PCOSQ and HQROL in PCOS women.
8	Hariprasath et al., 2023	•PCOSQ .<br>•Survey (additional questionnaire to measure "social impact").	No	N/A.	One-on-one interview, group discussion, and questionnaire.	Descriptive statistics.	PCOS and HRQOL, infertility, and social issues.
9	Hollinrake et al., 2007	•Primary Care Evaluation of Mental Disorders Patient Health Questionnaire (PRIME-MD PHQ).<br>• Beck Depression Inventory.<br>•Demographic questionnaire.	No	•Unable to evaluate the effects of PCOS on future risk of depression, because the study was cross-sectional in design.	In-person questionnaires.	Two-tailed t-test or Wilcoxon rank sum test. A chi-squared or Fisher exact test, logistic regression analysis, Pearson correlation, multiple linear regression analysis.	PCOS and depression, hyperandrogenism, and metabolic markers.
10	Hussain et al., 2015	Mini-International Neuropsychiatric Interview (MINI, English version 5.0.0).	No	•No community data in Kashmir on prevalence of psychiatric morbidity in the general population—difficult to generalize/compare data.	Interviews via psychiatrist.	Descriptive statistics; chi-squared tests	PCOS and psychiatric disorders (MDD, dysthymia, panic disorder, OSC, suicidality, BPD, GAD).

11	Jedel et al., 2008	PCOSQ Swedish version.	No	•Nonidentical settings in which surveys were taken and too many days between interferes with test–retest reliability.	Self-administered surveys.	Kappa (k) statistic with the intraclass correlation coefficient (ICC), one-way random-effects analysis of variance technique, Spearman rank-order correlations.	PCOSQ and HRQOL in Swedish PCOS women.
12	Jedel et al., 2010	•Comprehensive Psychopathological Rating Scale for Affective Syndromes (CPRS-S-A) *following 2 scales extracted.<br>•Brief Scale for Anxiety (BSA-S).<br>• Montgomery Asberg Depression Rating Scale (MADRS-S).	No	•Small sample size.<br>•Possible nonsignificant differences between cases and controls.<br>•Larger proportion of the women in the control group were students and therefore may be more likely to engage in regular physical exercise, thus confounding results due to the relationship between physical exercise and mood.	Self-reported computerized questionnaire.	Paired Student's t-test or Wilcoxon signed-rank test, conditional exact logistic regression models.	PCOS and anxiety and depression.
13	Jones et al., 2004	PCOSQ, SF-36.	Yes	•Face validity decreased after some women reported the questionnaires were not addressing acne concerns.<br>•Ratio of respondents to items was not large enough.<br>•Limited testing of construct validity because lack of similar questionnaires.<br>•Patients were recruited from a gynecology clinic. Possible bias towards PCOS patients with menstrual disturbance and infertility and not those with other PCOS symptoms.	Mailed paper questionnaires.<br><br>Face validity checked by interviewing 12 women with PCOS.	Secondary factor analysis, Cronbach's alpha statistic, intraclass correlation coefficients, item–total consistency, Wilcoxon signed-rank test, Spearman's nonparametric coefficient.	PCOSQ and HRQOL.

14	Joshi et al., 2021	•Beck's Depression Inventory.<br>•Hamilton Depression Rating Scale (HDRS).<br>•Hamilton Anxiety Rating Scale (HARS).<br>•Body Image Concern Inventory (BICI).<br>•Rosenberg' s Self-Esteem Scale (RSES).	Yes	•Selection bias because only patients from tertiary care center included.<br>•Did not use standard diagnostic criteria for diagnosing the prevalence of depression and anxiety.	Self-administered questionnaires.	Descriptive statistics with frequency distribution, Pearson's correlation coefficient. Two-tailed P values.	PCOS and depression, anxiety, body image disturbance, and self-esteem.
15	Karjula et al., 2017	Hopkins Symptoms Checklist-25 (HSCL-25).	Yes	•Self-reported PCOS and previous depression diagnosis, loss to follow-up.	Mailed questionnaires.	Logistic regression, Kruskal–Wallis H-test, Wilcoxon test.	PCOS and anxiety and/or depression symptoms, high BMI, or hyperandrogenism.
16	Karjula et al., 2020	15D Quality-of-Life Questionnaire, Hopkins Symptoms Checklist-25 (HSCL-25).	Yes	•15D tool enables comparison between cases and controls but not between women with PCOS, some loss to follow-up.	Mailed questionnaires.	Logistic regression.	PCOS and BMI, hyperandrogenism, socioeconomic status, HRQoL.
17	Karjula et al., 2021	Social Anhedonia Scale, (SAS), Physical Anhedonia Scale (PHAS), Perceptual Aberration Scale (PAS), Hypomanic Personality Scale (HPS), Bipolar II Scale (BIP2), and Schizoidia Scale (SCHD).	Yes	•Self-reported PCOS diagnosis, some scales had statistically significant findings while others did not, small sample size measuring for schizophrenia affects generalizability, homogenous population of white Caucasian women affects generalizability.	Paper-based surveys.	Cox regression analysis.	PCOS and psychosis, BMI, hyperandrogenism, and parental history of psychosis.
18	Klipstein et al., 2006	Mood Disorders Questionnaire (MDQ).	Yes	•Selection bias, self-report screen that may be less sensitive in detecting bipolar II disorder, lack of direct comparison with a control group, lack of knowledge of specific diagnostic workup for PCOS, lack of consensus regarding PCOS diagnostic criteria.	Mailed questionnaires.	Mann–Whitney and Fisher's exact tests; descriptive statistics and chi-squared analyses.	PCOS and bipolar disorder.
19	Kocak et al., 2022	PCOS Quality-of-Life Questionnaire (PCOSQ-50) and Beck Depression Inventory (BDI).	Yes	•Reporting bias, generalizability.	Paper-based surveys.	Shapiro–Wilk normality test.	PCOS and depression, socioeconomic status.

20	Kolahi et al., 2015	Dermatology Life Quality Index (DLQI) and Carver Coping Questionnaire.	Yes	•Reporting bias.	Paper-based surveys.	Kolmogorov–Smirnov test, Kruskal–Wallis, Mann–Whitney, Pearson correlation coefficient, and regression analysis	PCOS and age (>35 years old), employment status (self-employed worker), BMI, marital status.
21	Kumarapeli et al., 2010	World Health Organization Quality-of-Life Questionnaire (WHOQoL-BREF) and General Health Questionnaire (GHQ30).	Yes	•Generalizability.	Interviewer-administered questionnaire.	Kolmogorov–Smirnov test, Tukey's test, ANOVA, multiple linear regression.	PCOS and psychological distress.
22	Lam et al., 2005	1990 and 2003 Rotterdam criteria.	Yes	•Some women that were diagnosed with PCOS did not meet the criteria for both, selection bias.	Paper-based surveys and laboratory assays.	Logistic regression.	N/A.
23	Lee et al., 2022	Survey.	Yes	•Selection bias, many PCPs were not familiar with the Rotterdam criteria, small sample size.	Telephone interviews.	Thematic analysis.	N/A.
24	Lerchbaum et al., 2013	Measurements and laboratory assays.	Yes	•Selection bias, generalizability.	Paper-based surveys, patient physical exam assessment, and laboratory assays.	Descriptive statistics, Kolmogorov–Smirnov test, Kruskal–Wallis test, Mann–Whitney U test, k-index. and chi-squared test.	PCOS and prediabetes.
25	Lin et al., 2016	Chinese version of the Polycystic Ovary Syndrome Health-related Quality-of-Life Questionnaire (Chi-PCOSQ) and World Health Organization Quality-of-Life–BREF (WHOQOL--BREF).	Yes	•Self-reporting bias.	Paper-based surveys.	Paired t-tests, SRM, and confirmatory factor analysis.	N/A.
26	Maleki et al., 2022	Sexual Quality of Life—Female (SQOL-F) questionnaire, the Hurlbert Index of Sexual Compatibility, Hirsutism Questionnaire, and General Health Questionnaire (GHQ-28).	Yes	•Self-reporting bias.	Paper-based surveys.	Descriptive statistics, bivariate Pearson correlations, and a path model.	PCOS and sexual quality of life.
27	Mei et al., 2022	SF-36v2 and Mal-Polycystic Ovary Syndrome Questionnaire (Mal-PCOSQ).	Yes	•Self-reporting bias, generalizability.	Paper-based surveys.	Logistic regression and linear regression.	N/A.

28	Mojahed et al., 2023	Persian versions of BDI (Beck Depression Inventory), FSFIS (Female Sexual Function Index), and Sexual Quality of Life—Female (SQOL-F) questionnaires .	Yes	•Self-reporting bias, generalizability.	Paper-based surveys.	Linear regression.	PCOS and depression, sexual quality of life.
29	Nasiri-Amiri et al., 2016	PCOSQ-50 and the Short-Form Health Survey (SF-36).	Yes	•Generalizability, recall bias.	In-depth interviews and paper questionnaire.	Exploratory factor.	PCOS and quality of life, sexual function, income level.
30	Nasiri-Amiri et al., 2018	PCOSQ-50 questionnaire to obtain information on the health-related quality of life for PCOS patients and symptom distress.<br><br>Hayashi acne severity scale to measure acne.<br><br>Ferriman–Gallwey Scale to measure hirsutism.	Yes	•Cronbach's alpha and intraclass correlation coefficient for the coping subscale of the PCOSQ-50 were 0.61 and 0.5.	Surveys were collected through face-to-face interviews and standard questionnaires.<br><br>Physical exams were performed by trained midwives.	Kaiser–Meyer–Olkin and Bartlett test of sphericity to measure adequacy of sample size.<br><br>Confirmatory factor analysis and exploratory factor analysis were used to examine factor structure of PCOSQ-50.<br><br>Chi-squared, relative chi-squared, goodness-of-fit index, comparative fit index, normed fit index, root mean square error of approximation, and the standardized root mean square residual tests were used to assess the goodness-of-fit of the model.<br><br>Cronbach's alpha coefficient and item–total correlations were used to measure the internal consistency of the PCOSQ-50 questionnaire.<br><br>Test–retest method to assess the stability of	PCOS and health-related quality of life.

						the PCOSQ-50 questionnaire.	

31	Neubronner et al., 2021	Demographic survey to obtain the demographic information.<br><br>Reproductive health questionnaire to obtain the reproductive health information.<br><br>mGF score to measure hair growth.	No	•Women having undergone hair removal procedures may impact results of the mGF score measurement.	Surveys were collected via in-person surveys, transvaginal ultrasounds of the ovaries, and blood sampling. Hair growth was measured based on reference photographs by one of two investigators.	Independent sample t-test to detect differences in numerical variables.<br><br>Nonparametric Mann–Whitney U test was performed if the former did not have normality and homogeneity assumptions were not satisfied.<br><br>Pearson chi-squared test for categorical variables.<br><br>General linear modeling to compare the participant categories.<br><br>Bonferreoni corrections for pairwise comparison of means.<br><br>Logistic regression with odds ratio and p-value for binary outcomes regarding average menstrual cycle length.	PCOS individual clinical, ovarian, hormonal, and metabolic features and BMI.

32	Ning et al., 2013	Web-based questionnaire to obtain the clinics' demographic features and measures used to diagnose PCOS.	Yes	•Differentiation between 'professional' and 'nonprofessional' between survey respondents was not possible.<br><br>•The centers that entered data volunteered on their own and so there may be a selection bias.<br><br>•Multiple responses from the same clinic may have been included.<br><br>•The survey was subject to inconsistencies in respondent answers.<br><br>•Some of the practice patterns mentioned in questions did not focus the context on PCOS, and thus, answers may have been reported based on diagnosing other conditions.<br><br>•The series of yes-or-no questions were inadequate to fully encapsulate the practice pattern of an entire clinic.	Surveys were collected via an open-access internet-based survey tool that was posted on the IVF-Worldwide website.	Modified Wald method was used to calculate binomial confidence intervals for proportions.	Diagnostic criteria and PCOS diagnosis.

33	Ou et al., 2015	Chi-PCOSQ—the Chinese version of PCOSQ that looks at the health-related quality of life in PCOS patients.<br><br>WHOQOL-BREF and EQ-50—questionnaires used to look at health-related quality of life but not specific for PCOS patients.	Yes	•The CHI-PCOSQ was not validated with a large sample size nor for those with various education levels and socioeconomic statuses.<br><br>•Translation of PCOSQ into Chi-PCOSQ was performed by translators who were non-native English speakers.	Surveys were collected from participants via meeting with a well-trained interviewer.	Test–retest method to assess properties of PCOSQ and WHOQOL-BREF.<br><br>Cronbach's α values to assess internal reliability for each domain in PCOSQ, WHOQOL-BREF, and Chi-PCOSQ.<br><br>Comparative fit index to assess construct validity for WHOQOL-BREF.<br><br>Pearson correlation for test–retest reliability of Chi-PCOSQ.	PCOS and health-related quality of life in Chinese speaking women.
34	Panico et al., 2017	SCL-90-R and SF 36 were used as standardized psychometric questionnaires.<br><br>PCOSQ to measure the health-related quality of life and PCOS symptom distress.	Yes	N/A.	Surveys were collected via administering the questionnaires.	Pearson's coefficient to interpret the correlation between BMI and each of the test domains.<br><br>Student's t-test to verify the significance of the differences in the main score for each domain between PCOS and the healthy group, PCOS obese and PCOS lean patients, and PCOS obese patients and healthy obese controls.	PCOS, obesity, and health-related quality of life.

35	Patil et al., 2022	Ferriman–Gallwey score to measure hirsutism.	No	N/A.	Surveys were collected via detailed record forms, anthropometry, pelvis and abdominal ultrasonography, blood samples, biochemical tests, and clinical assessments for physical markers and for obvious anxiety or depression.	Categorical data were summarized as frequencies and percentages.<br><br>Continuous variables were summarized as means with standard deviations.	PCOS and its manifestations and comorbidities.
36	Patten et al., 2023	The Depression Anxiety Stress Scale (DASS-21) to measure depression, anxiety, and stress.<br><br>36-item Short-Form Health Survey (SF 36) to measure health-related quality of life.<br><br>PCOSQ to measure health-related quality of life and PCOS symptom distress.<br><br>Trial no.: ACTRN12615000242527.	Yes	N/A.	Surveys were collected via administering the surveys and obtaining blood samples and height/weight via in-person measurement.<br><br>Aerobic capacity was measured via an incremental maximal graded exercise test conducted on an electronically braked cycle ergometer, and it also included an electrocardiogram to screen for cardiovascular risks.	Linear mixed models to determine the effect of exercise intensity group over time and to determine the interaction between timepoint and group (between-group differences).<br><br>Estimated marginal means (from the linear mixed model) to examine the within-group changes.<br><br>Linear regressions to determine the association between peak oxygen uptake, insulin sensitivity, or body composition and mental health or health-related quality-of-life outcomes.<br><br>Mean ± standard deviation are used for the data.<br><br>Percentiles are used for boxplots.	PCOS with HIIT exercise training and mental health/health-related quality of life.

37	Petkova et al., 2018	PCOSQ to measure the health-related quality of life and PCOS symptom distress.	Yes	N/A.	Surveys were administered via pharmacies in Sofia, Bulgaria.	Factor group impact scores were obtained for each survey question.	PCOS and health-related quality of life in Bulgarian women.
38	Prathap et al., 2018	Ferriman–Gallwey score to measure hirsutism.<br><br>Hamilton Rating Scale for depression.<br><br>Hamilton Anxiety Rating Scale for anxiety.<br><br>WHO QOL-BREF surveys for quality of life.	No	•The Hamilton Anxiety Rating Scale had no minimum cut-off value.	Surveys were collected via administering the questionnaires.	Frequency and percentage were used for categorical variables.<br><br>Mean ± standard deviation for continuous variables.<br><br>Independent sample t-test, Mann–Whitney U test, Kruskal–Wallis test, and Spearman rank-order correlation coefficient were all used for normal and nonnormal data.	PCOS and anxiety, depression, and quality of life.
39	Radwan et al., 2023	Thirty-item electronic questionnaire to obtain the constructs (sociodemographic information, past medical and reproductive health history, and junk food consumption history).	No	•The survey was administered via a phone call and therefore may be subject to recall and social desirability biases.	Surveys were collected via administering the survey via telephone where the participants were contacted via a phone call and asked to respond to the questionnaire items.	Frequencies and percentages were used to express categorical values, and median and interquartile ranges were used to express continuous variables.<br><br>Multiple-response analysis was used to analyze variables within multiple sections of the questionnaire.<br><br>A Fisher’s exact test or Pearson’s chi-squared test was used to assess the association between PCOS symptoms and different patterns of fast food consumption.	PCOS and junk food consumption.

40	Rasgon et al., 2005	Ferriman–Gallwey score to measure hirsutism.<br><br>In-person questionnaire for past medical, reproductive, psychiatric, and family history.	No	•Reliance on self-report with regard to menstrual functioning can lead to women over-reporting or misattributing symptoms.	Surveys were collected via blood samples, physical exam, and in-person interviews with clinicians.	The binomial test was used to test comparisons between the frequencies of menstrual abnormalities, hirsutism, and acne.<br><br>The chi-squared test was used to assess the independent relationship between type of current medication and other clinical characteristics to the presence of menstrual abnormalities and to the development of new menstrual abnormalities. It was also conducted to estimate the prevalence of abnormal laboratory values of clinical significance.<br><br>T-tests to compare hormone levels between the groups.<br><br>Correlational analyses between BMI and levels of specific hormones, as well as between the duration of exposure to valproic acid and hormonal values.<br><br>A 3-group ANOVA to contrast between the groups (no valproate, valproate only, valproate + another medication).	Bipolar disorder and menstrual abnormalities, hormonal/metabolic abnormalities, or PCOS.

41	Robinson et al., 2020	Baseline questionnaire for maternal history, family history, and sociodemographic information.<br><br>Strengths and Difficulties Questionnaire (SDQ) and Vanderbilt ADHD Diagnostic Parent Rating Scale (VADPRS) for the mothers to rate their children's behaviors.<br><br>Annual questionnaire for the mother pertaining to the children's development.	Yes	•Self-reported bias based on the baseline questionnaire given to the mothers.<br><br>•Nonresponse to questionnaire follow-up.	Surveys were collected at baseline with the mothers completing the written questionnaires. When the children were 7 years old, the mothers completed the annual questionnaires regarding their children's development. The SDQ was completed when the children were 7 years old, and the VADPRS was completed when they were 8 years old.	Risk ratios and 95% confidence intervals were estimated for dichotomous end points using Poisson regression. These analyses were conducted using generalized estimated equations with standard errors to account for the correlation amongst twins.<br><br>Inverse probability weighting was used to account for nonresponse to the follow-up questionnaires. These were calculated from a multivariable logistic regression model.<br><br>A sensitivity analysis was conducted to account for self-reported maternal PCOS.<br><br>Associations were compared based on observed prevalence in the study with estimated associations of simulated samples for each of the misclassification errors.	Maternal self-reported PCOS and hirsutism and children's behavioral problems and mental disorders.

42	Rodrigues et al., 2012	Self-Reporting Questionnaire 20-Item Scale (SRQ scale-20) to measure common mental disorders.	Yes	•The study design required the reverse causality bias—so it was not possible to distinguish which condition manifested first based on the questionnaire, PCOS or the common mental disorder.	Surveys were collected at different clinics with specialized gynecology departments in Brazil where they had their height/weight taken, and patients filled out the sociodemographic survey and self-reporting questionnaire.	Chi-squared test was used to verify differences in proportions.<br><br>T-test was used to observe differences in means.<br><br>A chi-squared analysis was conducted after BMI categorization to check for differences in indication proportions of common mental disorders among women with and without PCOS.	PCOS and common mental disorders.
43	Rodriguez et al., 2020	Irregular cycle feature on the Clue app.	No	• Small sample size.<br>• Lack of clinical validation as it has not yet been tested on human subjects, thus results are not diagnostic.<br>• Cannot make predictions for individuals who have other disorders that cause menstrual cycle irregularity altering disorders or use hormone-based medications.<br>• Modeled to have high sensitivity and low specificity, attempts to send more people to receive screenings but will have false positives.	Clue app irregular cycle online feature.	Bayesian network, Pearson correlation coefficient, linear regression.	Correlation between responses of the irregular cycle feature and physician.

44	Rzo'nca et al., 2018	World Health Organization Quality-of-Life Scale (WHOQOL-BREF), Satisfaction With Life Scale (SWLS). and a standardized interview questionnaire.	Yes	• Only evaluated sociodemographic factors that affect QoL based on the WHOQOL-BREF questionnaire.<br>• Study did not analyze the impact of PCOS symptoms or psychological conditions on patients' life and functioning, thus did not evaluate health-related QoL.	Questionnaires and diagnostic surveys sent by mail.	Shapiro–Wilk test, Mann–Whitney U test, Kruskal–Wallis test, Cohen's d, Glass's delta, and chi-squared test ($\chi 2$).	Associations between quality of life and participant characteristics (socioeconomic standing, BMI, age, professional activity, children, and time from PCOS diagnosis) in PCOS patients versus in controls.
45	Salva-Pastor et al., 2020	Transient elastography Fibroscan and clinical exam.	No	• Lack of liver biopsies.<br>• Invasive nature of measurement tool.<br>• Possible sampling error.<br>• Small sample size.	In-person clinical exam.	Kolmogorov–Smirnov test, Student's t-test, and a multivariate logistic regression analysis.	NAFLD prevalence and severity in women with PCOS and controls.
46	Sánchez-Ferrer et al., 2017	Health questionnaires, gynecological and obstetrical history, physical and gynecological examination (including TVUS and blood draw), transvaginal ultrasound, time-resolved electrochemiluminiscence immunoassay, anogenital measurements.	No	• Possibility of confounding variables.<br>• Limitations in ability to determine causal relationships, observational study design.<br>• Possibility of selection bias.<br>• Possibility of information bias.	In-person health screening, gynecological exam, and questionnaire.	Unpaired Student's T- or Mann–Whitney U tests, chi-squared test, multiple linear regression analysis, unconditional multiple logistic regression.	Association between AGD measurements and PCOS. Associations between AGD measurements, reproductive hormone levels, number of antral ovarian follicles. Presence of PCOS phenotypic subtypes and AGD.
47	Santos et al., 2022	Initial health screening, filled out a medical history questionnaire, a Physical Activity Readiness Questionnaire (PAR-Q), and a short version of the International Physical Activity Questionnaire (IPAQ), Medical Outcomes Study Short-Form 36 (SF-36) questionnaire, and Depression, Anxiety, and Stress Scale (DASS-21).	Yes	• Small sample size.<br>• Lack of control of participants' diet.<br>• No reports of psychiatric comorbidities.<br>• Intensity levels of HIIT sessions were set based on a formula that determined maximum heart rate rather than a parameter derived from a maximal incremental test or employing heart rate reserve.	In-person health screening and questionnaire.	Shapiro–Wilk test and z scores, independent t-test or Fisher's exact test, normal Q-Q graph, and by the Akaike information criterion, Cohen's d, intention-to-treat, and per-protocol analyses.	Improvement in functional capacity, physical role functioning, general health perception, anxiety, and depression. With HIIT intervention.

48	Sari et al., 2020	Questionnaire Form, KSADS-PL semistructured interview, Rosenberg Self-Esteem Scale (RSES), Children's Depression Inventory (CDI), and Body Image Scale (BIS).	Yes	• Small sample size.<br>• Limitations in ability to determine causal relationships due to cross-sectional study design.<br>• Limited generalizability to large adolescent population.<br>• Did not include questions about future concerns of infertility.	In-person interview and questionnaire.	Kolmogorov–Smirnov test, Student's t-test, X2 test, Fisher exact test, and Pearson correlation coefficient.	Presence of psychiatric disorders in PCOS vs. control groups. Whether HOMA-IR (used as a marker of insulin resistance) was associated with psychiatric disorders in the PCOS group. Correlation of the modified FGS and total testosterone levels with RSES, CDI, and BIS scores.
49	Sayyah-Melli et al., 2015	Minnesota Multiphasic Personality Inventory (MMPI) and Primary Care Evaluation of Mental Disorders Patient Health Questionnaire (DSM-IV).	Yes	• Possible selection bias.<br>• Limited equal access to subjects.<br>• Some participants refused referral to clinical psychologist after recognition of suspected psychopathology.	In-person questionnaire.	Descriptive statistics, a chi-squared test, independent t-test, Wilcoxon–Mann–Whitney U tests, and Cramer's V.	Comparison of categorical variables and the difference between their means. Compare continuous variables. Rank the anxiety levels, depression scores, and other psychological disorders between groups. Severity of coefficient association for psychopathologic disorders.
50	Scaruffi et al., 2014	Rorschach Test and Millon Clinical Multiaxial Inventory-III (MCMI-III).	Yes	• Lack of rigorous PCOS diagnostic criteria decreases the ability to accurately diagnose the condition, which could lead to the inclusion of patients with conditions or characteristics that could affect the study or assessment of PCOS.<br>•Lack of precise diagnostic criteria for PCOS could have led to a decreased accuracy of diagnoses, subsequently impacting the understanding or identification of psychological disorders associated with PCOS.<br>•Small sample size.	In-person clinical exam and questionnaire.	t-test, ANOVA, Mann–Whitney U test, and Wilcoxon paired rank test.	Comparison of glycometabolic and hormonal data between PCOS and control groups. Distribution of nonparametric values. Com-<br>parison of the BR points from the<br>MCMI-III. Comparison of of the Rorschach indices responses from PCOS and control groups.

51	Scaruffi et al., 2018	Short-Form Health Survey (SF-36), the Attachment Style Questionnaire (ASQ), the Difficulties in Emotion Regulation Scale (DERS), the Toronto Alexithymia Scale (TAS-20), the Body Uneasiness Test (BUT), and the Minnesota Multiphasic Personality Invintory-2 (MMPI-2).	Yes	• Small sample size.<br>• Limited generalizability.<br>• Limitations in ability to determine causal relationships due to cross-sectional study design.<br>• Data were collected by self-reported measures.<br>• Factors such as hormonal levels, specific symptoms (e.g., hirsutism, infertility), and symptom severity were not examined in the study, potentially limiting the understanding of psychological factors associated with PCOS.	In-person questionnaire.	Descriptive statistics, t-tests, and Pearson chi-squared, all 2-tailed.	Mean differences across PCOS and control group demographic and clinical data.
52	Shakil et al., 2020	Demographic Information Form, Female Sexual Function Index (FSFI), Siddiqui Shah Depression Scale (SSDS), and Life Satisfaction Subscale (LSSS), clinical assessment, abdominal ultrasound, and a hormonal assay.	Yes	• Sample bias due to recruitment.<br>• Hospital cooperation in data collection.<br>• Small sample size.<br>• Lack of control group.<br>• Limited generalizability to fertile PCOS patients.<br>• Limitations in ability to determine causal relationships due to cross-sectional study design.	In-person clinical exam and questionnaire.	Regression analysis, Pearson's product moment correlation analysis.	Link between sexual dysfunction with depressive symptoms and life satisfaction.
53	Shaman et al., 2017	Menstrual history, clinical examination, transvaginal ultrasound examination, and biochemical blood analysis.	No	None mentioned.	In-person clinical exam.	Independent samples t-test, chi-squared, and conditional logistic regression.	Association between the presence of PCOS and the development of MS.

54	Shishehgar et al., 2016	Short-Form Health Survey 36 (SF 36).	Yes	• Inadequate power for subgroup analysis of various PCOS phenotypes.<br>• Generic HRQOL tool used rather than more sensitive, obesity-specific tools and specific questionnaires for assessing HRQOL in women with PCOS.<br>• Case-control design has inherent limitations for establishing causality between excess weight and HRQOL.<br>• Did not measure androgen levels or use ultrasonography for selected controls, potentially missing eumenorrheic nonhirsute women with less severe forms of PCOS.	In-person questionnaire.	Pearson's product moment correlation analysis, one-sample Kolmogorov–Smirnoff test, chi-squared, Bonferroni-adjusted univariate ANOVA, multivariate analysis of variances (MANOVA), univariate analyses and post hoc tests, multivariate analyses of covariance (MANCOVAs), and Fisher's Z test.	Differences in categorical variables among the study groups, differences in the SF-36 measures among each demographic group, differences in multidimensional scales of the SF-36, with study groups as the between-group factor and BMI groups, age, parity, FG scores, and menstrual regularity as covariates.
55	Sidra et al., 2019	Short-Form-12 Questionnaire. Evidence-based clinical checklist.	Yes	• Inability to test long-term effects of drugs.<br>• Inability to determine BMI due to lack of patient height data.<br>• Insufficient funding to test levels of biochemical parameters.	In-person questionnaire.	Chi-squared test, post hoc analysis.	Poor QOL scores are significantly associated with depression, comorbidities, hirsutism. Diarrhea and vomiting as the most common ADRs are significantly associated with the use of metformin, whereas uterine bleeding, weight changes, and reduced libido are significantly associated with the use of progestin. Patients receiving letrozole are significantly associated with higher QOL scores compared with those receiving clomiphene.
56	Smith et al., 2021	Genetic and Environmental PCOS Outcome Survey.	No	• Hypothetical study.<br>• Community sample might not reflect actual healthcare settings.<br>• Only intention was measured, not actual behavior.	Online survey.	Binary logistic regression, stratified sensitivity analyses.	Genetic causal explanations are significantly associated with secondary outcomes but not with environmental explanations.

57	Talpur et al., 2023	Self-reported questionnaire on PCOS outcomes.	No	• Patients' lack of knowledge.<br>• Social constraints and financial burdens.<br>• Conflicting outlook of PCOS.<br>• Ethnic disparities in biochemical and clinical characteristics of PCOS.	Online survey.	Chi-squared test.	Diagnosis of PCOS is significantly associated with those patients with primary infertility. Women affected by PCOS are significantly associated with an age younger than 35 compared to those of older age.
58	Varadan et al., 2019	Demographic and clinical parameters using Kei Satoh's method and Matheson et al. method.	Yes	• Out of MDA and MPO (oxidative stress parameters), only MDA showed a significant difference between PCOS and healthy subjects.<br>• Greater gingival inflammation was observed more in PCOS patients than the controls.	Clinical exam.	t-test, Pearson correlation coefficient test, and multiple linear regression analysis.	Women of young age (23-26 years old) are significantly associated with the frequency of gingivitis/mild periodontitis seen more dominantly in comparison to moderate/severe periodontitis. High GCF levels of MDA are significantly associated with PCOS patients compared to healthy patients.
59	Varanasi et al., 2018	YFHI questionnaire and Safe-D questionnaire.	Yes	• Only NIH criteria were applied due to lack of access to transvaginal ultrasound as per Rotterdam and AES criteria.<br>• Anthropometric data may not reflect participant's accurate body composition due to (5) site visits completed four years prior to supplementary questionnaire.<br>• Self-reported PCOS not confirmed by medical records.<br>• Small sample size.<br>• Serum androgen levels unavailable for 16% of participants, limiting the application of NIH criteria.	Online survey.	Shapiro–Wilk normality test, Mann–Whitney *U* tests, Fisher's exact, x^2 tests, multiple logistic regression, and thematic analysis.	Presence of acne is significantly associated with those participants who fulfilled NIH criteria compared to those who did not. Psychological comorbidity is significantly associated with self-reported diagnosis compared with not diagnosed.

60	Vutyavanich et al., 2007	Self-report survey based on the NIH criteria, the NIH criteria + polycystic ovary by ultrasonography, and NIH criteria + laboratory findings of hyperandrogenemia.	Yes	• Hospital-based study might not be representative for Northern Thai females.<br>• Focus on only one subgroup of Rotterdam PCOS criteria.<br>• Discrepancies in complaints associated with PCOS due to ethnic differences.<br>• Inability to measure free testosterone levels and testosterone index due to cost.<br>• Subjective diagnosis of hyperandrogenism.<br>• No BMI data on all participants.<br>• PCOS subjects not screened for metabolic syndromes.	In-person questionnaires.	Independent *t*-tests.	Infertility is more significantly associated with married PCOS subjects than with those who were not. Abnormal uterine bleeding and infertility are more significantly associated with PCOS than with hirsutism.
61	Wang et al., 2023	Hospital database.	No	• Small sample size.<br>• TCM tongue pulse PCOS diagnostic technique has yet to be reported.<br>• Time constraints.	Secondary analyses from a hospital database.	t-tests, Mann–Whitney U tests, AUROC, DeLong test, decision curve analysis, calibration curve, logistic LASSO regression.	TC-L and TC-a values were lower and TC-b was higher in the PCOS group than the health group. The differences in tongue data are statistically significant. TCM theory demonstrated tongue characteristics such as moistness and brightness are significantly associated with the difference between PCOS and healthy women. Low indicators of pulse condition are significantly associated with women with diagnosis. High risk of hypertension was also more significantly associated with PCOS than with healthy subjects.
62	Yan et al., 2021	Self-reported questionnaire based on the Rotterdam criteria, NIH criteria, and AE-PCOS criteria.	Yes	• Limited period of time for data collection.<br>• Small sample size.<br>• Lack of knowledge from OB/GYNs in identifying PCOS criteria.<br>• Unreliability of medical equipment.	Online survey.	Multivariate logistic regression.	Re-OB/GYNs from tertiary hospitals who saw more than 200 PCOS patients annually are significantly associated with the frequent use of Rotterdam criteria. Ge-OB/GYNs with less than 5 years of PCOS treatment, seeing less than 50 of those

							patients annually, are significantly associated with the use of AE-PCOS criteria. Rotterman criteria use is significantly associated with the use of oligomenorrhoea, biochemical, and clinical hyperandrogenism as diagnostic items but not with other diagnostic criteria. PCOS-related work experience is significantly associated with correct application rate of diagnostic criteria.
63	Zhang et al., 2012	Clinical assessments and laboratory tests.	No	• Ethnic differences.<br>• Variability in clinical characteristics and biochemical features.<br>• Cultural and lifestyle differences.	Clinical exam.	ANCOVA, *t*-tests, chi-squared tests.	Oligomenorrhea and polycystic ovarian presence was significantly associated with common PCOS phenotypes. Elevated levels of TT, increased ovarian volume, ovarian follicle numbers, longer menstrual cycle, and rate of acne are significantly more common in women with PCOS than those without. High LH levels were significantly associated with nonobese PCOS group than in obesity PCOS group.

Table S2. Type of methodology used and limitations of measurement tools.
